# Broad sarbecovirus neutralization by combined memory B cell antibodies to ancestral SARS-CoV-2

**DOI:** 10.1016/j.isci.2024.110354

**Published:** 2024-06-22

**Authors:** Cyril Planchais, Ignacio Fernández, Benjamin Chalopin, Timothée Bruel, Pierre Rosenbaum, Maxime Beretta, Jordan D. Dimitrov, Laurine Conquet, Flora Donati, Matthieu Prot, Françoise Porrot, Delphine Planas, Isabelle Staropoli, Florence Guivel-Benhassine, Eduard Baquero, Sylvie van der Werf, Ahmed Haouz, Etienne Simon-Lorière, Xavier Montagutelli, Bernard Maillère, Félix A. Rey, Pablo Guardado-Calvo, Hervé Nozach, Olivier Schwartz, Hugo Mouquet

**Affiliations:** 1 Institut Pasteur, Université Paris Cité, INSERM U1222, Humoral Immunology Unit, 75015 Paris, France; 2 Institut Pasteur, Université Paris Cité, Structural Virology Unit, 75015 Paris, France; 3 CNRS UMR3569, 75015 Paris, France; 4 CEA, INRAE, Medicines and Healthcare Technologies Department, SIMoS, Université Paris-Saclay, 91190 Gif-sur-Yvette, France; 5 Institut Pasteur, Université Paris Cité, Virus & Immunity Unit, 75015 Paris, France; 6 Centre de Recherche des Cordeliers, INSERM, Sorbonne Université, Université de Paris, 75006 Paris, France; 7 Institut Pasteur, Université Paris Cité, Mouse Genetics Laboratory, 75015 Paris, France; 8 Institut Pasteur, Université Paris Cité, G5 Evolutionary Genomics of RNA Viruses, 75015 Paris, France; 9 National Reference Center for Respiratory Viruses, Institut Pasteur, 75015 Paris, France; 10 Institut Pasteur, Université Paris Cité, Molecular Genetics of RNA Viruses, 75015 Paris, France; 11 Institut Pasteur, Université Paris Cité, CNRS UMR 3528, Cristallography Platform-C2RT, 75015 Paris, France

**Keywords:** immunology, virology

## Abstract

Antibodies play a pivotal role in protecting from SARS-CoV-2 infection, but their efficacy is challenged by the continuous emergence of viral variants. In this study, we describe two broadly neutralizing antibodies cloned from the memory B cells of a single convalescent individual after infection with ancestral SARS-CoV-2. Cv2.3194, a resilient class 1 anti-RBD antibody, remains active against Omicron sub-variants up to BA.2.86. Cv2.3132, a near pan-Sarbecovirus neutralizer, targets the heptad repeat 2 membrane proximal region. When combined, Cv2.3194 and Cv2.3132 form a complementary SARS-CoV-2 neutralizing antibody cocktail exhibiting a local dose-dependent synergy. Thus, remarkably robust neutralizing memory B cell antibodies elicited in response to ancestral SARS-CoV-2 infection can withstand viral evolution and immune escape. The cooperative effect of such antibody combination may confer a certain level of protection against the latest SARS-CoV-2 variants.

## Highlights

• Cv2.3194 is a resilient class 1 RBD SARS-CoV-2 neutralizer active up to BA.2.86
• Cv2.3132, a broadly sarbecovirus neutralizer, targets the HR2 membrane proximal region
• Combined Cv2.3132 and Cv2.3194 cooperate to neutralize SARS-CoV-2 variants *in vitro*
• Cv2.3132 and Cv2.3194 provide protection against SARS-CoV-2 infection in mouse models

## Graphical abstract

**Figure:**
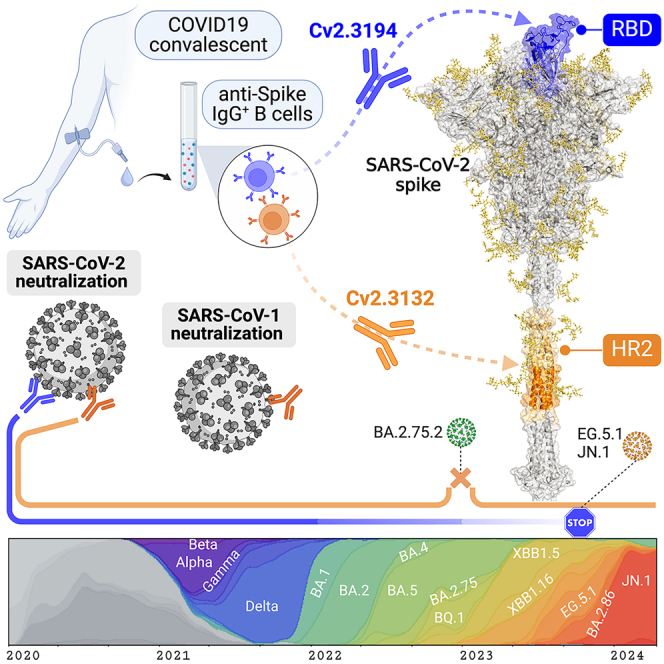

## Introduction

Neutralizing antibodies are paramount in protecting vaccinated and convalescent individuals from SARS-CoV-2 infection and re-infection.^1^^,^^2^^,^^3^ SARS-CoV-2 neutralizing monoclonal antibodies were also used effectively as prophylactic and therapeutic drugs against COVID-19.^4^^,^^5^ SARS-CoV-2 neutralizing antibodies target distinct sites on three major regions of the viral spike glycoprotein: the receptor binding domain (RBD), the N-terminal domain (NTD) and the S2 stalk.^6^^,^^7^ Neutralizing antibodies to the spike head (RBD and NTD) generally block directly or indirectly interactions with angiotensin-converting enzyme 2 (ACE2) receptor, while those to the S2 region interfere with viral fusion mechanism.^7^ Anti-RBD antibodies are often potent neutralizers,^7^ and belong to at least six classes depending on their epitope location either within (class 1 and 2) or outside (class 3 to 6) the receptor binding motif (RBM).^8^ However, the antigenic drift caused by continuous evolution of SARS-CoV-2 escaping immune pressure at a population level^9^^,^^10^ progressively dampened pre-existing humoral immunity relying on neutralizing antibodies.^3^ Viral escape to antibody neutralization was particularly marked with the emergence of Omicron lineages (BA.1 to BA.5)^11^^,^^12^^,^^13^^,^^14^ and exacerbated with the evolution toward new sub-lineages (e.g., BA.2.75/BA.2.75.2, BQ.1/BQ.1.1, XBB.1/XBB.1.15).^15^^,^^16^^,^^17^ Evasion from anti-RBD neutralizers due to mutations of interacting residues indeed culminated with the most recent Omicron sub-variants (15–27 RBD substitutions) such as EG.5.1 and FLip variants (i.e., HK.3), BA.2.86 and JN.1.^17^^,^^18^^,^^19^^,^^20^ Most monoclonal antibodies used in clinics also lost their neutralizing efficacy against SARS-CoV-2 variants, especially class 1 and 2 anti-RBD neutralizers.^21^ In contrast, sotrovimab (S309), a class 3 RBD-specific antibody, was more resilient and still neutralized, at a high concentration, the latest variants of concern (VOC) up to EG.5.^17^^,^^22^ Next-generation, broader, anti-RBD antibodies such as SA55, BD56-1854, BD57-0129, and Omi-42^15^^,^^20^^,^^23^^,^^24^ and more potent anti-S2 pan-SARS-CoV-2 neutralizers^25^ have also been reported.

We previously cloned two class 1 anti-RBD antibodies from memory B cells elicited in response to infection with ancestral SARS-CoV-2 and harboring potent neutralizing activities up to BA.2.^26^ Here, we examined the antiviral activities of those SARS-CoV-2 neutralizers, Cv2.1169 and Cv2.3194, against post-BA.2 VOC. We found that epistatic F486 mutations fixed in Omicron variants from BA.4/BA.5 completely shut down Cv2.1169 neutralization capacity. In contrast, Cv2.3194 still neutralized *in vitro* all VOC up to BA.2.86 and showed prophylactic activity against SARS-CoV-2 and BA.5 infection in mice. Strikingly, Cv2.3194-derived donor also developed a broadly neutralizing antibody targeting the heptad repeat 2 (HR2) S2 region, Cv2.3132, which when associated with Cv2.3134 in an antibody cocktail led to a cooperative neutralizing effect. Our data support the notion that combined resilient anti-RBD and anti-HR2 neutralizing antibodies, produced in response to ancestral SARS-CoV-2 infection, procured some level of protective immunity against VOC circulating after two years of viral evolution.

## Results

### BA.4- and BA.5-neutralizing capacities of class 1 anti-RBD Cv2.1169 and Cv2.3194

**Figure 1:**
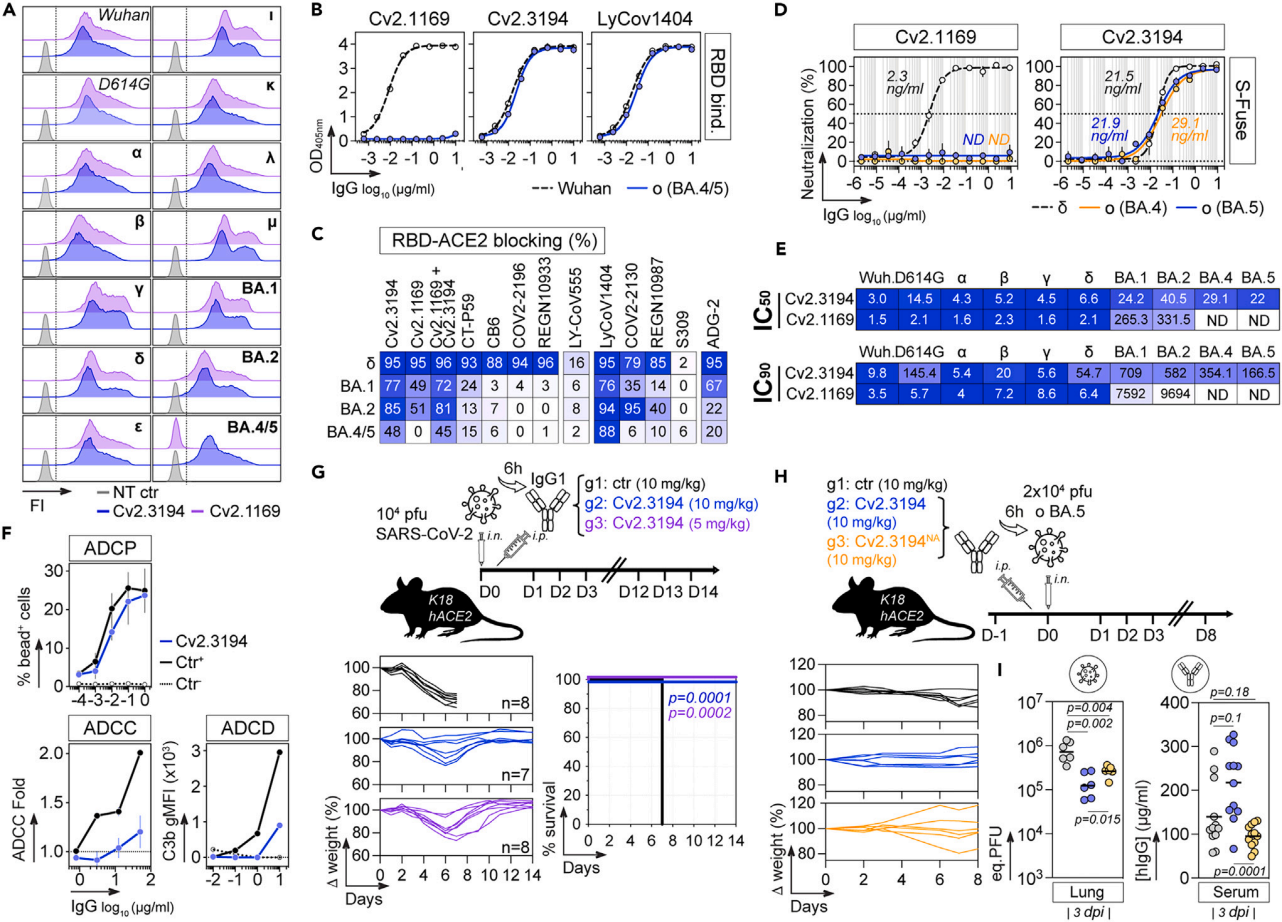
*In vitro* and *in vivo* neutralizing activity of Cv2.3194 against SARS-CoV-2 BA.5 (A) Representative histograms comparing the binding of Cv2.1169 and Cv2.3194 to the cell-expressed SARS-CoV-2 spike proteins as measured by flow cytometry. FI, fluorescence intensity. NT ctr, non-transfected cell control. (B) ELISA graphs comparing the binding of Cv2.1169 and Cv2.3194 to Wuhan and Omicron (o) BA.4/BA.5 RBD proteins. LyCov1404 was used as positive control. Means ± SD of duplicate values are shown. (C) Heatmap comparing the RBD-ACE2 blocking capacity of Cv2.1169, Cv2.3194, and benchmarked neutralizing antibodies. Darker blue colors indicate high competition while light colors show moderate competition (white = 0, no competition). Means of duplicate values (% binding inhibition) are shown in each cell. (D) Graphs comparing the neutralizing activity of Cv2.1169 and Cv2.3194 against Omicron (o) BA.4 and BA.5 viruses as determined with the S-Fuse neutralization assay. Delta virus (δ) was used as control. Error bars indicate the SD of replicate values from 2 independent experiments. IC_50_ values are indicated on the graphs. ND, not determined. (E) Heatmap comparing IC_50_ and IC_90_ values (in ng/mL) of Cv2.1169 and Cv2.3194 for the *in vitro* neutralization of selected viruses as determined with the S-Fuse neutralization assay. (F) Graphs showing the Fc-effector function activities of Cv2.3194 determined using *in vitro* assays. Non-SARS-CoV-2 IgG antibody mGO53 was used as negative control (Ctr-). ADCP activity (top graph) was measured as the % primary human monocytes positive for tri-S-coupled fluorescent beads. ADCC activity was measured as fold change compared to mGO53 Ctr- (dotted line, value = 1) in the ADCC reporter bioassay. Error bars indicate the SD of duplicate values. ADCD was measured by flow cytometry as geometric mean fluorescence intensity (gMFI) for the C3b deposition signal on antibody-bound spike-expressing cells. Anti-RBD antibodies Cv2.1169 (for ADCP) and Cv2.6264 (for ADCC and ADCD) were used as positive controls (Ctr+). (G) Schematic diagram showing the experimental design of Cv2.3194 antibody therapy in SARS-CoV-2-infected K18-hACE2 mice (top). Animals were infected intranasally (i.n.) with 10^4^ plaque forming units (PFU) of SARS-CoV-2 and received 6 h later an intraperitoneal (i.p.) injection of Cv2.3194 or isotypic control IgG antibody at ∼ 10 mg/kg (0.25 mg) and ∼5 mg/kg (0.125 mg). Graphs showing the evolution of initial body weight (% Δ weight, bottom left) and survival rate (bottom right) in animal groups. Groups of mice were compared in the Kaplan-Meier analysis using Log rank Mantel-Cox test. (H) Schematic diagram showing the experimental design of Cv2.3194 antibody prophylaxis in BA.5-infected K18-hACE2 mice (top). Animals received an intraperitoneal (i.p.) injection of Cv2.3194, Cv2.3194 N297A mutant (Cv2.3194^NA^) or isotypic control IgG antibody at ∼ 10 mg/kg (0.25 mg) and were challenged intranasally (i.n.) 6 h later with 2 × 10^4^ PFU of BA.5. Graphs showing the evolution of initial body weight (% Δ weight) in animal groups are shown on the bottom left. (I) Dot plots comparing between animal groups the intra-lung RNA loads as equivalent PFU (eq. PFU) (left) and serum titers of administrated human IgG antibodies (hIgG) (right) measured at 3 dpi. Groups of mice were compared using two-tailed Mann-Whitney test. See also Figures S1 and S2.

To evaluate the activity of Cv2.1169 and Cv2.3194 against Omicron BA.4 and BA.5, we first tested their binding to cell-expressed spike by flow cytometry and purified recombinant RBD proteins by ELISA. Cv2.3194 but not Cv2.1169 strongly bound to the BA.4/5 spike and RBD protein (Figures 1A and 1B). In agreement, Cv2.3194 but not Cv2.1169 maintained RBD-ACE2-blocking and *in vitro* neutralizing activities against BA.4 and BA.5 (Figures 1C and 1D). This indicates that the F486V substitution in BA.4 and BA.5, when combined with pre-existing RBD mutations of antibody-interacting residues (K417N, S477N, T478K, and Q493R),^26^ completely abolishes SARS-CoV-2 recognition and neutralization by Cv2.1169. Indeed, the F486V mutation alone only moderately reduced the bivalent antibody binding of Cv2.1169 (Figure S1A), suggesting epistasis of RBD substitutions as previously described.^27^ In contrast, Cv2.3194 efficiently neutralized BA.4 and BA.5 in the S-Fuse assay with IC_50_ of 29.1 ng/mL and 21.9 ng/mL, and IC_90_ of 345 ng/mL and 166 ng/mL, respectively (Figures 1D and 1E). In addition, Cv2.3194 displayed a strong antibody-dependent cellular phagocytosis (ADCP) potential, but weak antibody-dependent cellular cytotoxicity (ADCC) and antibody-dependent complement deposition (ADCD) activities (Figure 1F and S1B–S1D).

**Figure 2:**
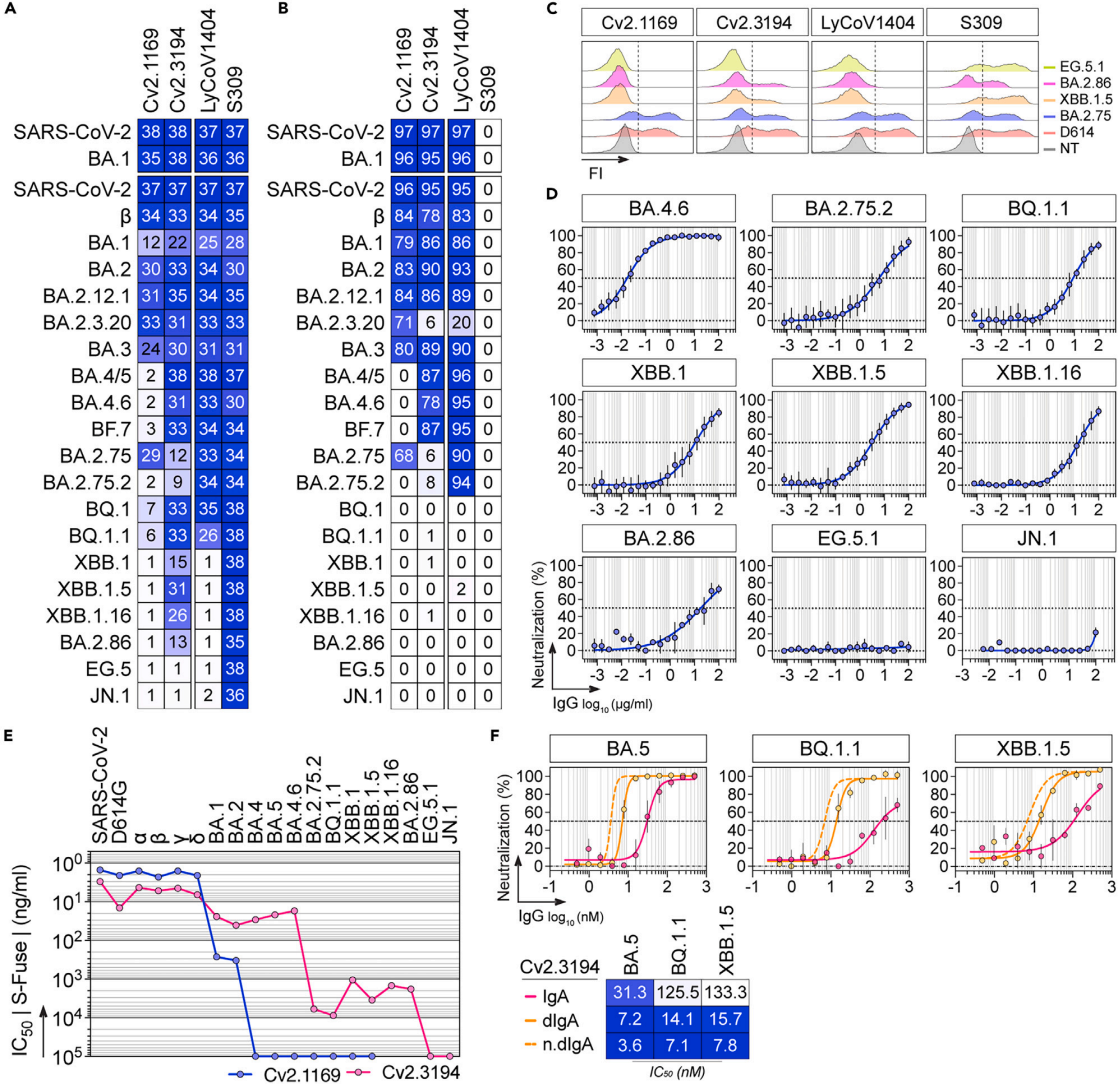
Binding and *in vitro* neutralizing capacities of Cv2.3194 against Omicron sub-variants (A) Heatmap showing the binding of Cv2.1169 and Cv2.3194 to the soluble recombinant tri-S (top) and RBD (bottom) proteins as measured by ELISA (mean AUC from duplicate titration values with a maximum antibody concentration of 10 μg/mL). Cells are color-coded according to area under the curve (AUC) values with darker colors indicating high binding while light colors show moderate binding (white = no binding). (B) Heatmap showing the tri-S- (top) and RBD-ACE2 blocking capacity of Cv2.1169 and Cv2.3194. Darker blue colors indicate high competition while light colors show moderate competition (white = 0, no competition). Means of duplicate values (% binding inhibition) are shown in each cell. (C) Representative histograms comparing the binding of Cv2.1169, Cv2.3194, S309, and LyCoV1404 (at 10 μg/mL) to the cell-expressed SARS-CoV-2 spike proteins as measured by flow cytometry. FI, fluorescence intensity. NT, non-transfected cell control. (D) Graphs showing the neutralization curves of Cv2.3194 against selected SARS-CoV-2 VOC as determined with the S-Fuse neutralization assay. Error bars indicate the SD of replicates values from 4 to 6 independent experiments. (E) Graphs comparing the IC_50_ values of Cv2.1169 and Cv2.3194 against SARS-CoV-2 VOC tested in the S-Fuse neutralization assay. Values from SARS-CoV-2 to BA.2 are historical data.^26^ (F) Graphs comparing the SARS-CoV-2 neutralizing activity of Cv2.3194 produced as monomeric and dimeric IgA (dIgA) antibodies against selected VOC as determined with the S-Fuse neutralization assay. Error bars indicate the SD of replicates values from 2 independent experiments. n.dIgA, normalized values according to the number of binding sites. Heatmap (bottom) presents the IC_50_ values calculated from the curves (top). See also Figures S1 and S2.

The *in vivo* therapeutic potential of Cv2.3194 was next evaluated using the K18-hACE2 transgenic mouse model for SARS-CoV-2 infection. Mice infected i.n. with 10^4^ PFU of SARS-CoV-2 (D614G strain) were treated 6 h later with a single i.p. injection of Cv2.3194 IgG antibody (0.125 mg, ∼5 mg/kg and 0.25 mg, ∼10 mg/kg) or control IgG antibody (0.25 mg, ∼10 mg/kg). Infected mice in the control group lost up to 25% of their body weight within the first 6 days post-infection (dpi) before reaching humane endpoints at 7–8 dpi, while all Cv2.3194-treated animals survived and recovered their initial body weight (Figure 1G). We then tested the prophylactic activity of Cv2.3194 and its N297A-mutated IgG version (Cv2.3194^NA^), which is unable to bind to Fcγ receptors (Figures S1B–S1D), against BA.5 infection in K18-hACE2 transgenic mice. Antibodies were administrated (10 mg/kg) once 6 h prior to infection with 2 × 10^4^ PFU of BA.5. In this model, infection with BA.5 is less severe than with the original strain. Thus, mice did not experience consistent weight loss and did not succumb to infection (Figure 1H). Therefore, we quantified by RT-qPCR the intra-lung viral content as equivalent plaque forming units (eqPFU), which was significantly reduced at 3 dpi in Cv2.3194-treated animals compared to controls (1.46 × 10^5^ vs. 8.07 × 10^5^ eqPFU/lung, *p* = 0.002) (Figure 1I). Cv2.3194^NA^ also decreased viral RNA loads (2.71 × 10^5^ eqPFU/lung, *p* = 0.004), but slightly less efficiently than Cv2.3194 (*p* = 0.015) (Figure 1I). Lower circulating antibody levels of Cv2.3194^NA^ detected in mouse sera (95.73 vs. 217.56 μg/mL, *p* = 0.0001) (Figure 1I), likely accounted for the difference of activity between the two Cv2.3194 versions. Yet, we cannot exclude that Fc-dependent effector functions may have participated to reducing pulmonary viral loads.

### Neutralization spectrum of Cv2.3194 against Omicron BA.2- and BA.4/5-derived sub-variants

Next, we evaluated the binding and neutralization capacities of Cv2.3194 against post-BA.5 Omicron sub-variants. Cv2.3194 bound strongly and comparably to all RBD proteins including newly tested variants (BA.2.12.1, BA.2.3.20, BA.3, BA.4.6, and BF.7), but showed a drastic reduction of reactivity against BA.2.75, BA.2.75.2, BQ.1, BQ.1.1, XBB.1.5, XBB.1.16, and BA.2.86, and did not bind EG.5 and JN.1 (Figures 2A, S2A, and S2B). In agreement, Cv2.3194 efficiently blocked RBD-ACE2 interactions by ELISA for SARS-CoV-2 variants except for BA.2.75, BA.2.75.2, BQ.1, BQ.1.1, XBB.1.5, XBB.1.16, and BA.2.86, which would certainly require much higher inhibitory antibody concentrations (Figures 2B and S2B). Moreover, Cv2.3194 still bound, albeit to a lesser degree, to BA.2.75, XBB.1.5, and BA.2.86 but not EG.5.1 spikes by flow cytometry (Figure 2C). Consistently, although Cv2.3194 neutralized authentic BA.2.75.2, BQ.1.1, XBB.1, XBB.1.5, XBB.1.16, and BA.2.86 viruses in the S-Fuse neutralization assay (Figure 2D), IC_50_ values were on average 2.2 log_10_ lower than for BA.5 (Figure 2E). Cv2.3194 did not neutralize EG.5.1 and JN.1 (Figures 2D and 2E). In contrast, Cv2.1169 was completely inactive against post-BA.2 variants (Figure 2E). Our published data showed that the avidity effect of the dimeric form of genuine Cv2.1169 antibodies could rescue its neutralization efficacy against BA.1 and BA.2.^26^ To test whether binding avidity could enhance the neutralizing activity of Cv2.3194 against less sensitive VOC, we produced recombinant monomeric and dimeric IgA versions of Cv2.3194 and compared their activity in the S-Fuse assay. In agreement, Cv2.3194 IgA dimers had 9-, 18- and 19-fold higher neutralizing potency against BA.5, BQ.1.1, and XBB.1.5, respectively, than their monomeric counterparts when normalized for the number of binding sites (Figure 2F).

### Epitope mapping and affinity maturation of Cv2.3194

**Figure 3:**
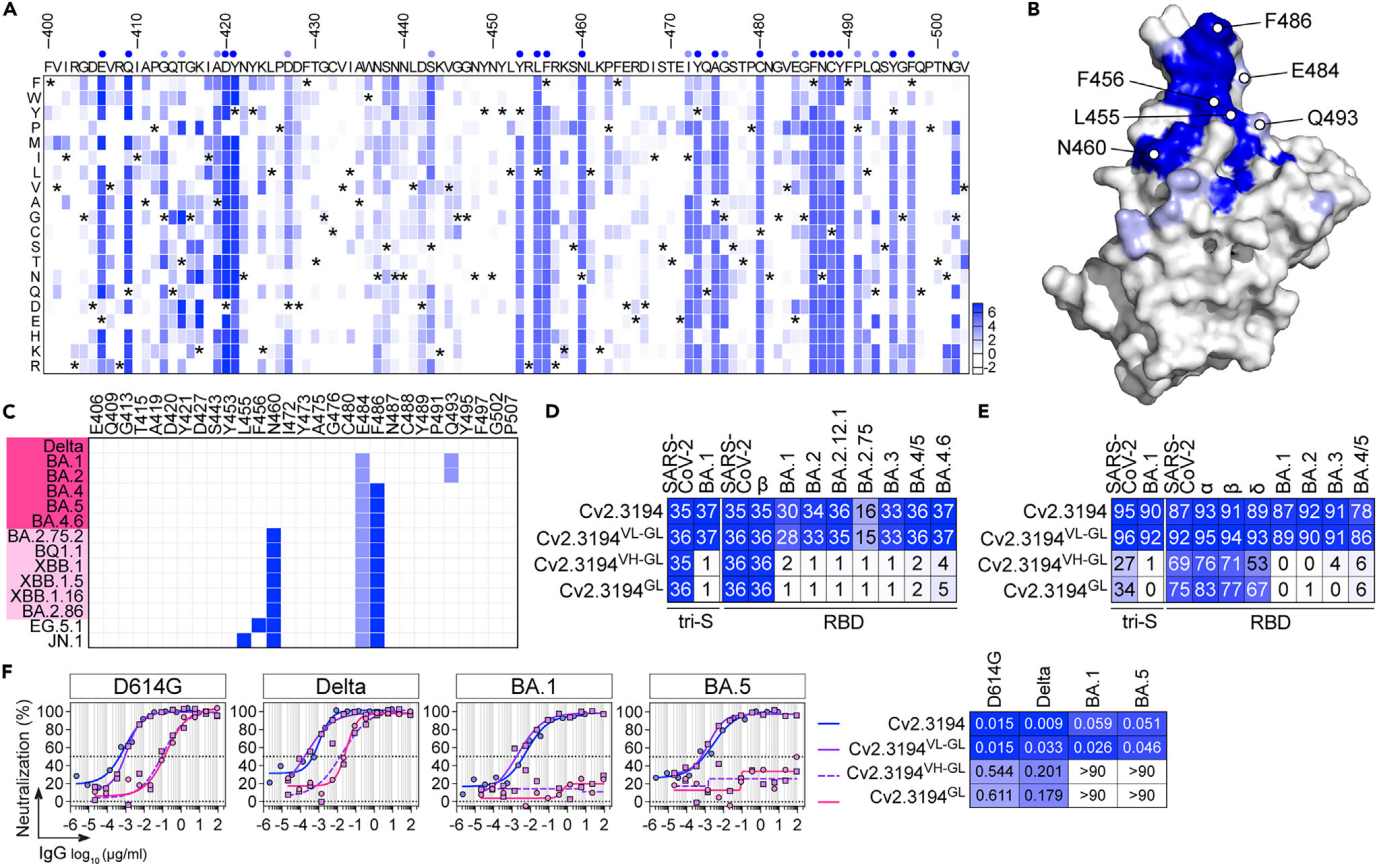
Epitope mapping and antibody maturation of Cv2.3194 (A) NGS-based deep mutational scanning (DMS) heatmap showing heatmap showing the enrichment scores of RBD single mutants after functional sorting by FACS using Cv2.3194 Fab as bait. Enrichment score is a log2 function of the frequency fold-change between sorted and unsorted RBD yeast populations for a given amino acid substitution. The corresponding heatmap is colored in blue for enriched mutations. The index is set as the number of substitutions with an enrichment score higher than 2. Asterisks indicate the original amino acid residues. (B) Surface representation of the SARS-CoV-2 RBD structure (PDB: 7QEZ) (gray) presenting the putative amino acid residues interacting with Cv2.3194 (light and dark blue) identified with the DMS analysis in (A). Dark blue color indicates residues for which 80% of the substitutions (*n* = 19) compromise Cv2.3194 antibody binding to RBD mutants. Among these residues, those mutated in BA.2.75.2, BQ.1.1, XBB.1/XBB.1.5, EG.5.1 and JN.1 variants are indicated. (C) Table showing the putative Cv2.3194-RBD interacting residues identified by DMS that are mutated on selected SARS-CoV-2 VOC (blue). Dark and light pink highlights indicate highly sensitive and sensitive VOC to Cv2.3194 neutralization, respectively; resistant viral variants are not colored. (D) Heatmap comparing the binding of parental Cv2.3194, germline reverted (Cv2.3194^GL^) and hybrid antibody variants (Cv2.3194^VH−GL^ composed of Cv2.3194 IgL/Cv2.3194^GL^ IgH and Cv2.3194^VL−GL^ composed of Cv2.3194 IgH/Cv2.3194^GL^ IgL) to the soluble recombinant tri-S and RBD proteins as measured by ELISA (mean AUC from duplicate values). Cells are color-coded according to area under the curve (AUC) values with darker colors indicating high binding while light colors show moderate binding (white = no binding). (E) Heatmap comparing the tri-S- and RBD-ACE2 blocking capacity of Cv2.3194, Cv2.3194^GL^ and hybrid antibody variants (Cv2.3194^VH−GL^ and Cv2.3194^VL−GL^). Darker blue colors indicate high competition while light colors show moderate competition (white = 0, no competition). Means of duplicate values (% binding inhibition) are shown in each cell. (F) Representative graphs comparing the *in vitro* neutralizing activity of Cv2.3194, Cv2.3194^GL^ and hybrid antibody variants (Cv2.3194^VH−GL^ and Cv2.3194^VL−GL^) against selected SARS-CoV-2 variants as determined with the S-Fuse neutralization assay. Heatmap presenting the IC_50_ values calculated from the neutralization curves obtained from 2 independent experiments is shown on the right. See also Figure S3.

To map the epitope of Cv2.3194, we first used a deep mutational scanning (DMS) based on the antibody binding to yeast-displayed RBD mono-mutants by flow cytometry. Cv2.1169, for which we obtained the X-ray crystal structure in complex with the RBD protein^26^ was also analyzed by DMS. Fab fragments of Cv2.1169 and Cv2.3194 bound with nanomolar apparent affinities to wild-type SARS-CoV-2 RBD expressed at the yeast surface (Figure S3A). DMS experiments were performed with two independent mutant libraries encompassing the RBD residues 383 to 512 (Bank #2 and #3; Figure S3B), and clones affecting the binding of Fabs were sequenced to uncover deleterious single mutations (Figure S3C). Mutations identified by DMS as altering Cv2.1169 recognition covered the RBD-interacting surface previously defined by structural analyses (Figures S3D and S3E), validating our methodological approach. For Cv2.3194, 29 substitutions (from position 406 to 507) decreased RBD reactivity, to various degrees depending on the replacing amino acid (Figure 3A). Collectively, these mutations define a putative binding fingerprint located in the RBM, straddling the RBD ridge and leaning toward the occluded face of the RBD when the domain is in down state (Figure 3B). Four potential Cv2.3194 contacting residues are mutated in Omicron variants: E484 and Q493, having a moderate effect on the RBD recognition by Cv2.3194, and N460 and F486, which caused a drastic reduction of binding when mutated (Figures 3B and 3C). In line with this, SARS-CoV-2 sub-variants with strongly reduced neutralization sensitivity, BA.2.75.2, BQ1.1, XBB.1, and XBB.1.5, combined N460K, E484A and F486V/F486S substitutions, whereas BA.1 and BA.2, both harboring E484A and Q493R mutations, and BA.4/5 combining mutated F484 and F486 residues, remained neutralized by Cv2.3194, albeit with a slight decrease in efficacy compared to pre-Omicron variants (Figures 2E and 3C). This indicates that the N460K substitution greatly alters SARS-CoV-2 binding and neutralizing activities of Cv2.3194. Additional RBD mutations of the putative Cv2.3194-contacting residue L455 and F456 as found in JN.1 and EG.5 variants conferred full resistance to antibody neutralization (Figures 2D, 3B, and 3C). Of note, the sole presence of the triple mutation F456A-N460K-F486V considerably reduced Cv2.3194 binding to the RBD protein (Figure S1A).

We next examined the impact of somatic mutations on the binding and neutralizing activities of Cv2.3194. The putative germline precursor (GL) of Cv2.3194 reacted with spike or RBD proteins from original and Beta strains but not to Omicron variants (Figure 3D). Accordingly, Cv2.3194^GL^ partially blocked tri-S/RBD-ACE2 interactions and neutralized SARS-CoV-2 and its variants up to Delta (Figures 3E and 3F). Pairing Cv2.3194^GL^ IgH with mutated IgL (Cv2.3194^VH−GL^) did not rescue SARS-CoV-2 Omicron binding and neutralization, whereas Cv2.3194^VL−GL^ combining mutated IgH with GL IgL conserved binding and neutralizing activities of parental Cv2.3194 (Figures 3D–3F). Together, these data indicate that Cv2.3194 recognizes an epitope in the RBM, bears V_H_ somatic mutations crucial for its activity against BA.1 to BA.5, and partially or completely loses its neutralization activity when viral variants acquired combined RBD mutations at positions L455 or F456, N460 and F486.

### Structural characterization of the Cv2.3194 epitope

**Figure 4:**
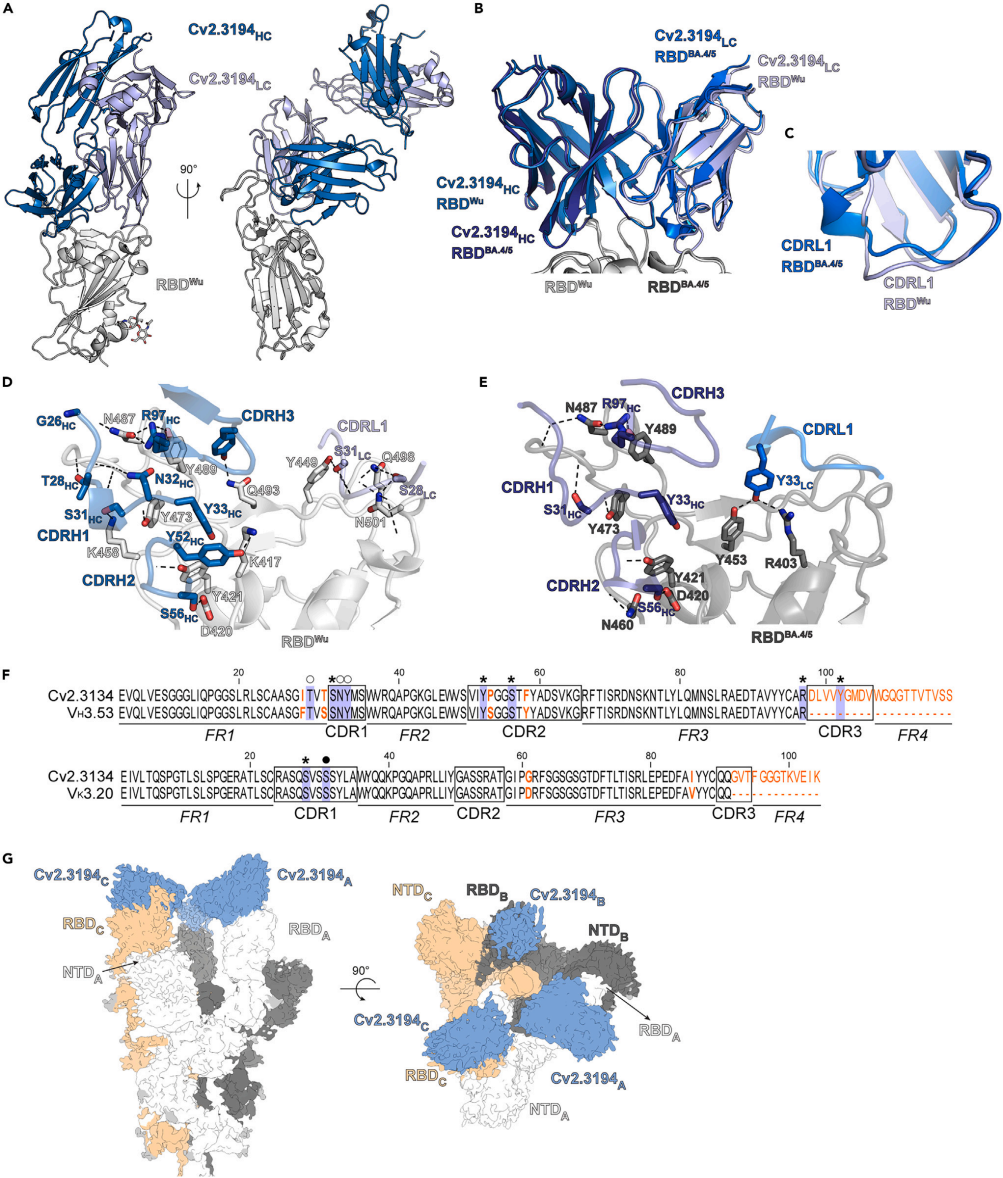
Structural analyses of the Cv2.3194 epitope (A) Crystal structure of the ancestral SARS-CoV-2 RBD (RBD^Wu^) in complex with Cv2.3194 Fab (PDB: 8QH0). Heavy chain (HC) and light chain (LC) are shown in dark blue and light blue, respectively. (B) Superimposition of the crystal structures obtained with RBD^Wu^ (light gray) and BA.4/5 RBD (RBD^BA.4/5^, dark gray) (PDB: 8QH1). The Fab bound to the Omicron RBD is indicated in darker shades. (C) Zoom into the superimposition shown in (B) highlighting the different conformation of the CDR_L_1 when Cv2.3194 Fab is in complex with RBD^Wu^ (light blue) or RBD^BA.4/5^ (dark blue). (D) Close-up at the Cv2.3194-RBD^Wu^ interface, with only the side chains from residues forming hydrogen bonds (dashed lines) from the CDRs are shown as sticks. (E) Close-up at the Cv2.3194-RBD^BA.4/5^ interface with only the side chains from residues forming hydrogen bonds (dashed lines) from the CDRs are shown as sticks. (F) Amino acid alignments of Cv2.3194 variable domain and germline V_H_3-53∗01 (top) and V_K_3-20∗01 (bottom) sequences. Complementary determining regions (CDR) and framework regions (FR) are indicated. Mutated residues are highlighted in bold and colored orange. In the alignment, Cv2.3194 residues contacting RBD^Wu^ (blue boxes) are marked as closed circles when their side chain interacts with RBD amino acid main and side chain, open circles for main chain only, and stars for side chains only. (G) Cryo-EM map from the Wuhan Spike ectodomain in complex with Cv2.3194 (EMDB: EMD-18399). The three Spike protomers are identified with subscripts (A, B, and C) and different colors. See also Figures S4 and S5, and Tables S1–S4.

To define the epitope of Cv2.3194 at atomic level and the antibody’s interactions with the RBM, we crystallized the Cv2.3194 Fab in complex with the RBD from ancestral SARS-CoV-2 (RBD^Wu^) and BA.4/5 (RBD^BA.4/5^) strains and determined their structures by X-ray crystallography. The Cv2.3194 Fab-RBD^Wu^ complex produced crystals diffracting to 1.9 Å, allowing us to refine an atomic model to a final *R*_*free*_ value of 0.21. The Cv2.3194 Fab-RBD^BA.4/5^ crystals diffracted to 2.65 Å, and we refined an atomic model to a final *R*_*free*_ of 0.23 (Table S1). The structure showed that the antibody binds to residues within the RBM and the buried face of the RBD (Figure 4A). The buried surface area (BSA) of Cv2.3194 upon binding is ∼1000 Å^2^, with the IgH variable domain predominantly contributing and the CDR_H_3 accounting for one-third of the IgH BSA (Table S2). Several features of Cv2.3194 including the ACE2 binding site overlap, the paratope BSA value, and the contribution of the different CDR_H_s are common with other class 1 V_H_3-53 antibodies.^28^ Structural alignment of the RBDs from the two crystallized complexes showed the same binding mode for Cv2.3194 (root-mean-square deviation of 1.1 Å across 1646 atoms corresponding to V_H_ and V_L_) (Figure 4B; Table S2). Most of the differences between the two structures were found in the CDR_L_1, which adopts a helical turn in the complex with RBD^BA.4/5^, moving slightly away from the RBD (Figures 4C and S4E). An extensive network of polar contacts is found at the interface with Cv2.3194 and the RBD^Wu^ (Figure 4D), including side chains from residues that are mutated in various VOC (i.e., K417, Q493, N501, Q498) (Tables S3 and S4). Cv2.3194 IgL variable domain formed fewer hydrogen bonds upon binding to RBD^BA.4/5^ (Tables S3 and S4), thus allowing conformational changes in the CDR_L_1. Alignments of Cv2.3194 variable domain and germline V_H_3-53∗01 and V_K_3-20∗01 amino acid sequences revealed the conservation of residues interacting with RBD^Wu^ (Figure 4F), providing a rationale behind SARS-CoV-2 neutralization by Cv2.3194^GL^ (Figure 3F). The residue N460 identified by DMS as a potential contacting residue for Cv2.3194 is at the periphery of the epitope, right in front of an electropositive patch on Cv2.3194’s paratope (Figure 4E). Thus, the N460K mutation introduces a positive charge inducing an electrostatic repulsion and a bulkier side chain difficult to accommodate in the interface (Figure S4A). As a result, this mutation reduced spike-binding affinity and SARS-CoV-2 neutralizing activity of Cv2.3194. The residue F486 was also buried at the epitope rim (Table S3), and mutations that reduced the residue surface, such as F486V in the Omicron variants, could further decrease the affinity of Cv2.3194 for the RBD. To provide a structural basis for the resilience of Cv2.3194, we performed a structural alignment of a series of V_H_3-53-encoded class 1 anti-RBD antibodies including Cv2.3235, which we previously characterized.^26^ Unlike these antibodies, which have a long CDR_L_3 that interacts with and constrains the CDR_H_3 and CDR_L_1 loops, the CDR_L_3 loop of Cv2.3194 is unusually short, allowing the formation of a cavity in the paratope (Figures S4B–S4D). We speculate that this cavity could enable the CDR_H_3 and CDR_L_1 to accommodate residue variations in different viral variants. Notably, comparison of the structures of Cv2.3194 in complex with RBD^Wu^ and RBD^BA.4/5^ revealed conformational differences at the CDR_L_1 level (Figures 4C and S4E), which would likely not be possible if the CDR_L_3 loop had rigidified the paratope.

We also determined the cryo-electron microscopy (cryo-EM) structure of the Cv2.3194 Fab in complex with a stabilized spike ectodomain at 3.2 Å (Figures 4G and S5A). The structure showed an open spike with the three RBDs in the “up” conformation (Figure 4G). The local resolution at the RBDs was lower than for the rest of the spike protein (Figure S5A), indicating that these domains are highly mobile in this conformation. The cryo-EM map presented extra densities on top of the RBDs, which was compatible with binding of the Fab (Figure 4G), and consistent with the RBD-Cv2.3194 crystal structure (Figure S5B). Since Cv2.3194 interacted with RBM residues on the occluded face of the RBD, it only recognized the RBD in the “up” position, locking this conformation and leading to the 3-up-RBD state of the spike we observed (Figure 4G). A distance ≤65 Å between the Cα of the IgH residue 222 from adjacent Fabs bound to the spike has been used to evaluate whether intra-spike crosslinking by an IgG is possible.^29^ Thus, we measured the distance between the Cα of the residues 219 (last residue modeled in the IgH of the crystal structure) of adjacent Cv2.3194 Fabs fitted into the cryo-EM map and obtained 46.1 Å, suggesting that both arms of the Cv2.3194 IgG can bind to a single adjacent spike protein leading to a stronger binding by avidity effects.

### Characterization of anti-HR2 SARS-CoV-2 neutralizer Cv2.3132

**Figure 5:**
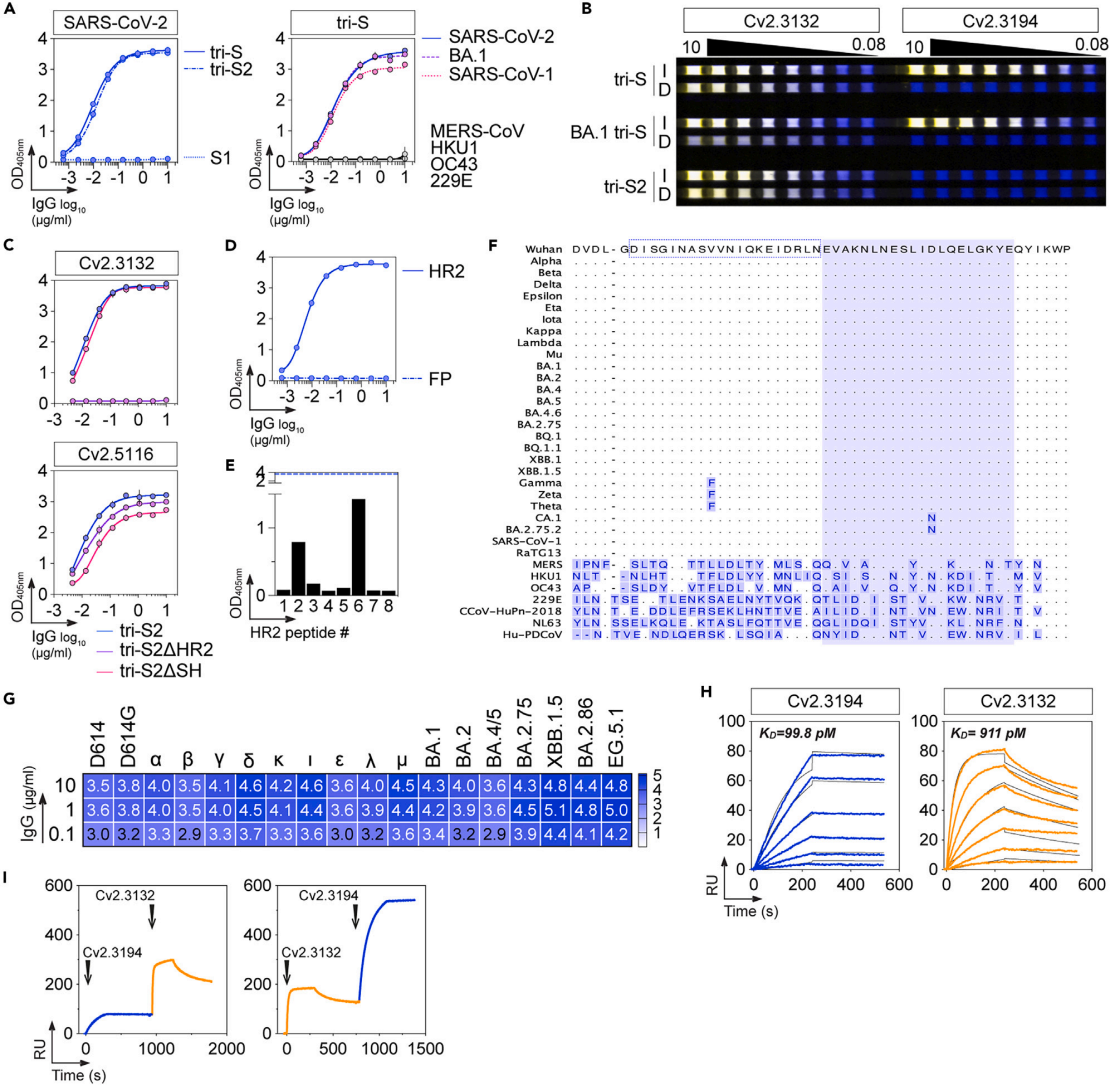
Binding characteristics of anti-HR2 antibody Cv2.3132 (A) Graph comparing the ELISA binding of Cv2.3132 IgG antibody to SARS-CoV-2 tri-S, tri-S2 and S1 proteins (left), and spike trimers from other coronaviruses (α-coronaviruses: 229E, and β-coronaviruses: OC43, HKU1, SARS-CoV-1, MERS-CoV) (right). SARS-CoV-2 BA.1 tri-S was also included. Means ± SD of duplicate values are shown. (B) Infrared dot blot comparing the reactivity of Cv2.3132 and Cv2.3194 antibodies (yellow) to intact (I) and denatured (D) SARS-CoV-2 tri-S, BA.1 tri-S, and tri-S2 proteins detected with an anti-histidine tag monoclonal antibody (blue). (C) Graph comparing the ELISA binding of Cv2.3132 IgG antibody (top) to SARS-CoV-2 tri-S2, tri-S2ΔHR2 and tri-S2ΔSH proteins. Anti-S2 antibody Cv2.5116 (bottom) was included as control. Means ± SD of duplicate values are shown. (D) Graph comparing the ELISA binding of Cv2.3132 IgG to FP (K22K) and HR2 peptides. Means ± SD of duplicate values are shown. (E) Graphs showing the ELISA reactivity of Cv2.3132 IgG against 20-mer HR2 overlapping 5-amino acid peptides (*n* = 8). Means ± SD of triplicate values are shown. (F) Amino acid alignment of the HR2 spike region (position 1163–1213) from selected *Coronaviridae* viruses. Mismatched residues are shown in blue. (G) Heatmap showing the flow cytometric binding of Cv2.3132 IgG to spike-expressing 293-F cells for SARS-CoV-2 variants (SARS-CoV-2, D614G, [VOC]: α, β, γ, δ, BA.1, BA.2, BA.4/5, BA.2.75, XBB.1.5, BA.2.86, EG.5.1, [VOIs]: ε, ι, κ, λ, μ). Geometric means of duplicate log_10_ ΔMFI values are shown in each cell. (H) SPR sensorgrams showing the relative affinity of Cv2.3194 and Cv2.3132 IgG antibodies for the binding to SARS-CoV-2 S trimers (tri-S). Calculated K_D_ values are indicated on the graphs. RU, response units. (I) SPR sensorgrams showing the binding of Cv2.3194 on Cv2.3132:tri-S complexes (left) and of Cv2.3132 on Cv2.3194:tri-S complexes (right). RU, response units. See also Figure S6.

**Figure 6:**
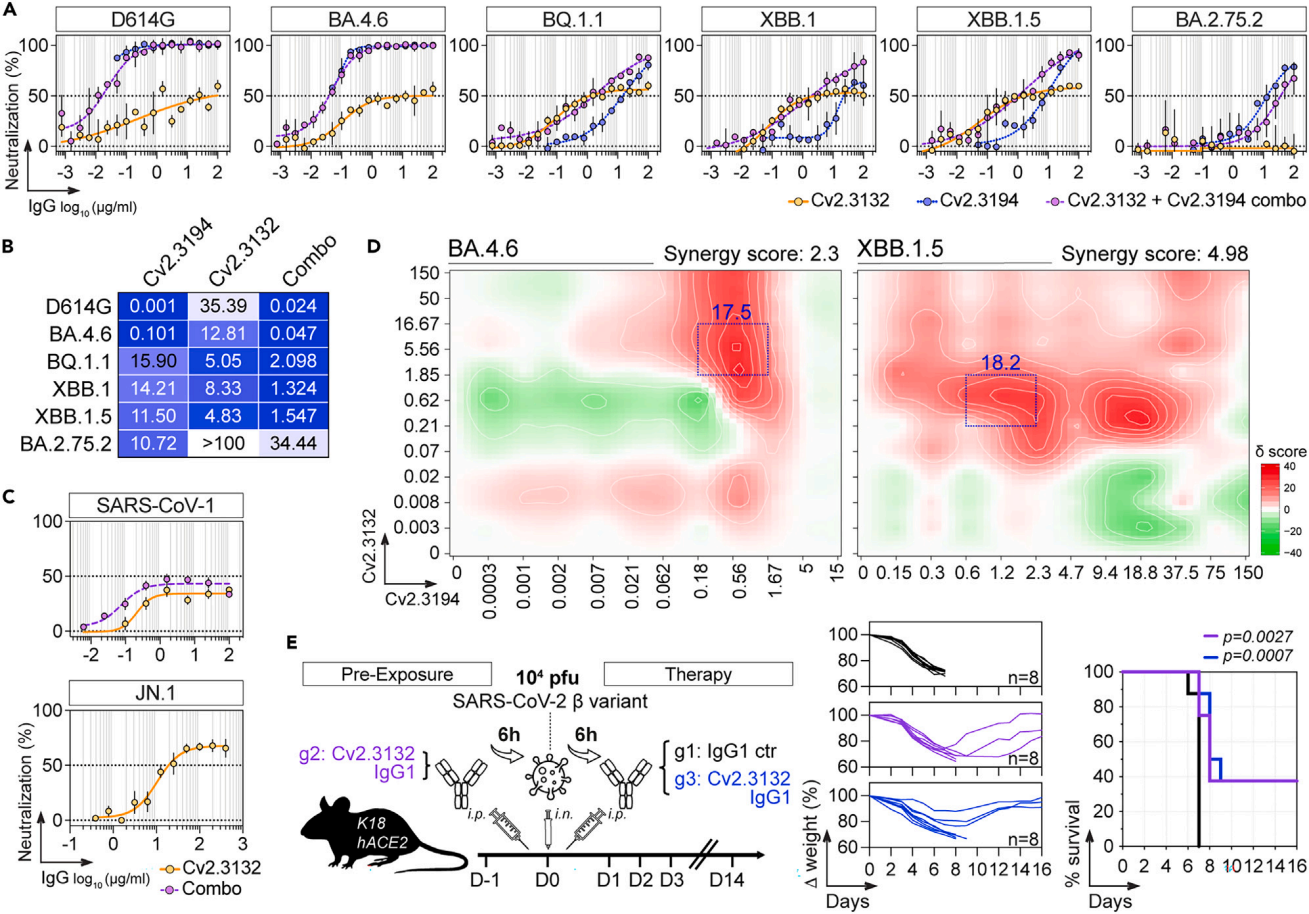
SARS-CoV-2 neutralizing capacities of Cv2.3132 (A) Graphs comparing the *in vitro* neutralizing activity of Cv2.3194, Cv2.3132 and combined antibodies (Cv2.3194 + Cv2.3132) against selected SARS-CoV-2 variants as determined with the S-Fuse neutralization assay. Error bars indicate the SD of values from 2 independent experiments. (B) Heatmap comparing the IC_50_ values (in μg/mL) for the SARS-CoV-2 neutralization calculated from (A) for Cv2.3194, Cv2.3132 and combined antibodies (Combo). (C) Graphs showing the *in vitro* neutralizing activity of Cv2.3132 and combined antibodies (Cv2.3194 + Cv2.3132) against SARS-CoV-1 as determined with the SARS-CoV-1 plaque-reduction neutralization test (PRNT) (top) and JN.1 as determined with the S-Fuse assay (bottom). Error bars indicate the SEM of triplicate values from 2 independent experiments for SARS-CoV-1 and of values from 3 independent experiments for JN.1. (D) Synergy maps calculated on the dose-response neutralization matrices of combined Cv2.3194 and Cv2.3132 against BA.4.6 and XBB.1.5 shown in (Figure S6D). Synergistic area are shown in red. Blue dotted square represent the highest synergy score (δ score). Global and highest local area synergy scores are indicated on each map. (E) Schematic diagram (left) showing the experimental design of Cv2.3132 antibody treatment in K18-hACE2 mice infected intranasally (i.n.) with 10^4^ PFU of the SARS-CoV-2 variant β (B.1.351). Animals were either pre-treated 6h before infection with ∼10 mg/kg (1 mg) of Cv2.3132 IgG or treated 6 h post-infection with ∼10 mg/kg (1 mg) of Cv2.3132 IgG or mGO53 isotype control (ctr) by intraperitoneal (i.p.) injection. Graphs showing the evolution of initial body weight (% Δ weight, middle) and survival rate (right) in animal groups. Groups of mice were compared in the Kaplan-Meier analysis using Log rank Mantel-Cox test. See also Figure S6.

From the Cv2.3194-derived convalescent donor (CC3), we also identified an IgG antibody with neutralization potential targeting the S2 spike region, termed Cv2.3132 (Figure 5A).^26^ Cv2.3132 cross-reacted with the recombinant spike trimers of SARS-CoV-1, but not of other α-and β-coronaviruses (Figure 5A).^26^ Cv2.3132 bound to a non-quaternary S2 epitope (Figure 5B).^26^ Cv2.3132 recognized recombinant trimeric S2 proteins deleted from the stem helix (SH) but not from the HR2 portion, which is located adjacent to the transmembrane domain (Figure 5C). It also reacted well with the HR2 peptide but not with the fusion peptide (FP) (Figure 5D). Mapping analysis with HR2-overlapping peptides showed that Cv2.3132 recognizes mainly, but weakly compared to the entire HR2 peptide (D1163-P1213), the peptide E1188-E1207 in the C-terminal portion of SARS-CoV-2 HR2 (Figures 5E and S6A). Thus, Cv2.3132 targets the HR2 membrane proximal region, which is a highly conserved region across Sarbecoviruses including SARS-CoV-2 variants (Figure 5F). In agreement, Cv2.3132 had comparable binding to spike-expressing cells from VOC α, β, γ, δ, BA.1, BA.2, BA.4/5, BA.2.75, XBB.1.5, BA.2.86, EG.5.1, and VOIs ε, ι, κ, λ, μ (Figures 5G and S6B). Cv2.3132 displayed high affinity binding to SARS-CoV-2 spike as measured by surface plasmon resonance (SPR) (K_D_ = 0.91 nM), with faster on and off rates than Cv2.3194 (*k*_*a*_ = 1.3 × 10^6^ vs. 7.4 × 10^5^ M^−1^s^−1^, *k*_*d*_ = 1.2 × 10^−3^ vs. 7.4 × 10^−5^ s^−1^, respectively) (Figure 5H). SPR analyses showed that Cv2.3194 and Cv2.3132 bind simultaneously to the spike protein without interfering with each other (Figure 5I). Cv2.3132 neutralized *in vitro* SARS-CoV-2 D614G (IC_50_ = 35.4 μg/mL), and the more recent BA.4.6, BQ.1.1, XBB.1, and XBB.1.5 SARS-CoV-2 variants with IC_50_ values ranging from 4.8 to 8.3 μg/mL in the S-Fuse assay but reaching a neutralization plateau at ∼ 50% (Figures 6A and 6B). BA.2.75.2 virus was resistant to Cv2.3132 antibody neutralization (Figure 6A), most likely due to the D1199N substitution in the HR2 C-terminal region of this variant (Figure 5F). Considering the location of Cv2.3132 epitope on the S2 subunit, and the inability of Cv2.3132 to block spike-ACE-2 interactions by ELISA (Figure S6C), we hypothesize that the antibody interferes with membrane fusion mechanism.

### Cooperative neutralization of Cv2.3194 and Cv2.3132

Cooperative and/or synergistic effects of antibody cocktails made of anti-RBD and anti-S2 (FP or stem helix) neutralizers have been reported.^25^^,^^30^^,^^31^^,^^32^ Therefore, we evaluated whether combining Cv2.3132 with Cv2.3194 would lead to additive or synergistic effects. First, we compared the *in vitro* neutralizing activity of an equimolar Cv2.3132-Cv2.3194 IgG mixture to single antibodies. We found that the Cv2.3132-Cv2.3194 combination exhibited superior neutralization than single antibodies against BA.4.6, BQ.1.1, XBB.1, and XBB.1.5 (IC_50_ range: 0.05–2.1 μg/mL) (Figures 6A and 6B). Combined antibodies did not neutralize BA.2.75.2 as efficiently as Cv2.3194 alone due to the lack of neutralizing activity of Cv2.3132 against this variant (Figures 6A and 6B). Cv2.3132 alone or combined with Cv2.3194 neutralized SARS-CoV-1 with a 40–50% plateau as observed for SARS-CoV-2 and remained also active against JN.1 (IC_50_ = 10.4 μg/mL) (Figure 6C). Synergy-scoring model analysis of *in vitro* neutralization experiments made with BA.4.6 and XBB.1.5 showed that although both antibodies did not globally act in synergy (total synergy δ-score of 2.3 and 4.98, respectively; while a global synergy δ-score would range from −10 to 10), high local synergistic effects were observed at certain concentrations (Figures 6D and S6D). Thus, for instance, highest δ-scores for XBB.1.5 neutralization were obtained in the range of 0.6–2.3 μg/mL and 0.21–1.85 μg/mL for Cv2.3194 and Cv2.3132, respectively (Figure 6D).

To determine whether Cv2.3132 is active *in vivo*, we tested its prophylactic and therapeutic activity against SARS-CoV-2 VOC Beta in K18-hACE2 transgenic mice as previously described.^26^ A single administration of Cv2.3132 IgG antibodies at ∼10 mg/kg (0.25 mg *i.p.*) 6 h prior to (pre-exposure) or after (therapy) infection with 10^4^ PFU of SARS-CoV-2 Beta (β) protected 37.5% of the animals from death (vs. 0% in the control group; *p* = 0.003 and *p* = 0.0007, respectively; Figure 6E).

Together, these data indicate that anti-HR2 antibody Cv2.3132 is a broad Sarbecovirus neutralizing antibody, active *in vivo*, and efficiently blocking SARS-CoV-2 infection with the most recent VOC when combined with the resilient class 1 anti-RBD antibody Cv2.3194 isolated from the same donor.

## Discussion

We report on the detailed molecular and functional features of a resilient SARS-CoV-2 neutralizing antibody, Cv2.3194, elicited in an individual infected during the first epidemic wave.^26^ We previously described Cv2.3194 as a potent class 1 anti-RBD neutralizing all VOC up to BA.2.^26^ Here, we found that Cv2.3194 progressively loses its potency on BA.2/BA.5 sub-variants and becomes inactive against EG.5.1 and JN.1 variants, which emerged in July–August 2023. Using an integrative experimental approach, we precisely defined the epitope of Cv2.3194 and showed that viral variants combining RBD mutations of the antibody’s interacting residues L455 or F456, N460, and N486 in the pre-existing Omicron backbone, fully abrogated Cv2.3194 binding and neutralizing activities. Thus, Cv2.3194 resisted complete immune escape from the viral evolution of SARS-CoV-2 spanning over 2 years and still neutralized, despite a lower efficacity, VOC accumulating up to ∼25 RBD-substitutions (e.g., XBB.1.16 and BA.2.86). We postulate that the uncommonly short CDR_L_1 contributes to the adaptability of the Cv2.3194 paratope in accommodating antigenic variations in viral variants. Consequently, such resilient V_H_3-53 class 1 neutralizer may have been rarely elicited in response to infection. Of note, a resilient V_H_1-58/V_K_3-20 class 1 RBD neutralizing IgA antibody that developed in response to ancestral SARS-CoV-2 infection has also been recently described.^33^ Spike-specific memory B cells are instrumental for a durable immune protection against SARS-CoV-2,^34^ and continue their affinity maturation by accumulating antibody somatic mutations for up to one year after acute infection.^35^^,^^36^^,^^37^ This memory B cell clonal evolution enables anti-RBD antibodies, including V_H_3-53/V_K_3-20 class 1 to which Cv2.3194 belongs, to gain neutralization breadth and potency.^38^ Hybrid immunity conferred by vaccination after infection, or vice versa, and/or heterotypic antigen exposure through re-infections with novel VOC may further drive the affinity maturation of some memory B cell antibodies toward greater neutralizing capacities.^39^^,^^40^ Cv2.3194 was cloned from CC3 donor early in convalescence and had very low hypermutation rates. It is thus plausible that its corresponding memory B cell lineage naturally evolved to adapt to newly encountered variants by giving rise to more potent and broad neutralizers, as observed for broadly HIV-1 neutralizing antibodies.^41^ Whether such scenario of clonal B cell evolution responding to viral escape truly occurred for Cv2.3194 lineage remains to be investigated. Apart from B cell adaptation, protecting against highly drifted viral strains in individuals with such B cell specificities would likely require repeated exposures to antigenically distant variants to override immune imprinting driven by ancestral strains.^42^ Monoclonal antibodies effective against currently circulating viral strains are still needed for pre-exposure prophylaxis in immunocompromised populations. Therefore, Cv2.3194 could be engineered to augment their affinity against post-BA.5 variants. Enhanced neutralization potency and breadth could be achieved through successive artificial affinity maturation cycles combining deep random-mutagenesis and functional screening steps. This approach was employed with ADI-55689 to produce ADG-2 (adintrevimab),^43^ and further-optimized antibody VYD222 (pemivibart), which is currently under evaluation in clinical trials^44^ and has just received FDA authorization for emergency use in the United States.

Out of only 19 anti-spike monoclonal antibodies identified in donor CC3,^26^ we found another broad SARS-CoV-2 neutralizing antibody, Cv2.3132, targeting a conserved HR2 epitope on the prefusion spike. Combining Cv2.3194 and Cv2.3132 did not lead to a strict functional synergy, but associating both antibodies in a cocktail generated a local concentration-dependent synergy for neutralizing SARS-CoV-2 VOC *in vitro*. Thus, the co-existence of complementary resilient class 1 anti-RBM and anti-HR2 neutralizers may provide some levels of protection against re-infection with viral variants. Cv2.3132 recognizes a region on the prefusion HR2 helical bundle that is likely disordered by the conformational changes and structural rearrangements necessary for transitioning to the postfusion form of the SARS-CoV-2 spike.^45^ Hence, Cv2.3132 epitope destabilized during the viral fusion process may be inaccessible on the post-fusion form. Although human antibodies targeting this region may be elicited upon infection with different coronaviruses,^46^ no genuine human monoclonal antibodies to the HR2 C-terminus were characterized so far. A panel of human anti-HR2 monoclonals were previously derived from Xenomice (bearing human immunoglobulin genes) immunized with the SARS-CoV-1 spike.^47^ In line with our results, combining Xenomouse-derived anti-HR2 and anti-RBD antibodies enhanced SARS-CoV-1 neutralization with a potential synergy.^47^ More recently, potent and broad SARS-CoV-2 HR2 neutralizing nanobodies cloned from a MERS-CoV/SARS-CoV-1/SARS-CoV-2 Spike-immunized lama have been described.^48^ One of these single-domain antibodies (VHH), R3DC23, recognizes a quaternary epitope on the membrane proximal region of HR2. Three R3DC23 VHH molecules, each binding to two adjacent helices in the HR2 3-helix bundle, block viral fusion through the potential locking of the HR2 coiled coil. R3DC23 also neutralized SARS-CoV-1, and protected hamsters from SARS-CoV-2 infection as a human IgG1-like engineered molecule.^48^ Cv2.3132 also neutralized SARS-CoV-1 but as for SARS-CoV-2 isolates, neutralization reached a plateau at approximatively 50%. This incomplete neutralization *in vitro* may explain why despite its potency the antibody protected *in vivo* about half of the infected animals. The neutralization mechanism of Cv2.3132 and the molecular basis for its partial neutralizing activity still need to be investigated. It is possible that the poor accessibility of the epitope stuck between N1192-branched glycans and the viral membrane and/or the steric hindrance for IgG binding preclude the full locking of a single HR2 bundle by 3 Cv2.3132 molecules, as observed for R3DC23,^48^ and subsequent complete viral neutralization.

In summary, we describe in detail two resilient broadly neutralizing memory B cell antibodies elicited in a COVID-19 convalescent donor in response to the ancestral SARS-CoV-2 infection. One targets the RBM, the other the HR2 region, making them compatible in a complementary association to neutralize a wide spectrum of betacoronaviruses and especially SARS-CoV-2 variants. These combined memory B cell lineages may provide a level of protection against re-infections with diverging SARS-CoV-2 variants, particularly due to their potential clonal evolution toward increased neutralization properties.

### Limitations of the study

In this study, while we identified the HR2 membrane proximal region of the SARS-CoV-2 spike as the target of Cv2.3132, we did not precisely delineate the epitope of Cv2.3132 at atomic level, nor did we unveil the molecular mechanisms of viral neutralization by Cv2.3132. This would be important to provide an explanation for the partial neutralization observed with Cv2.3132, but it remains to be investigated. Likewise, we did not fully elucidate the mechanistic details explaining the resilience of the V_H_3-53-expressing class 1 anti-RBD antibody Cv2.3194. Moreover, we did not trace the evolution of the Cv2.3194 antibody lineage following vaccination and/or subsequent VOC infection to determine whether antibody variants with enhanced activity against post-BA.5 viruses emerged.

## STAR★Methods

### Key resources table

**Table:** 

REAGENT or RESOURCE	SOURCE	IDENTIFIER
**Antibodies**		

Human monoclonal IgG1 mGO53	Wardemann et al.^49^	N/A
Human monoclonal IgG1 Cv2.1169	Planchais et al.^26^	N/A
Human monoclonal IgG1 Cv2.3194	Planchais et al.^26^	N/A
Human monoclonal IgA1 Cv2.3194	This study	N/A
Human monoclonal dimeric IgA1 Cv2.3194	This study	N/A
Human monoclonal Fab-IgG1 Cv2.3194	This study	N/A
Human monoclonal Fab-IgA1 Cv2.3194	This study	N/A
Human monoclonal IgG1 Cv2.3132	Planchais et al.^26^	N/A
Human monoclonal IgG1 S309	Planchais et al.^26^	N/A
Human monoclonal IgG1 COV2-2196	Planchais et al.^26^	N/A
Human monoclonal IgG1 REGN10933	Planchais et al.^26^	N/A
Human monoclonal IgG1 CB6	Planchais et al.^26^	N/A
Human monoclonal IgG1 CT-P59	Planchais et al.^26^	N/A
Human monoclonal IgG1 LY-CoV555	Planchais et al.^26^	N/A
Human monoclonal IgG1 COV2-2130	Planchais et al.^26^	N/A
Human monoclonal IgG1 REGN10987	Planchais et al.^26^	N/A
Human monoclonal IgG1 ADG20	Planchais et al.^26^	N/A
Human monoclonal IgG1 LyCoV1404	Westendorf et al.^50^	N/A
Goat anti-human IgG Alexa Fluor 647	Life technologies	#A-21445; RRID: AB_2535862
Donkey Anti-human IgG Alexa Fluor 680	Jackson ImmunoReseach	#709-625-149; RRID: AB_2340582
Goat anti-mouse IgG IR Dye® 800CW	LI-COR Biosciences	#NC9401841
Peroxidase AffiniPure Goat Anti-Human IgG, Fcγ fragment specific	Jackson ImmunoReseach	#109-035-098; RRID: AB_2337586
Mouse anti-Hisx6 antibody	BD Biosciences	# 552565; RRID: AB_10514425

**Bacterial and virus strains**		

SARS-CoV-2 D614G strain	National Reference Center for Respiratory Viruses	EPI_ISL_414631
SARS-CoV-2 B.1.351 strain	National Reference Center for Respiratory Viruses	hcoV-19/France/IDF-IPP00078/2021
SARS-CoV-2 BA.2.75.2 strain	Planas et al.^16^	EPI_ISL_15731524
SARS-CoV-2 BQ.1.1 strain	Planas et al.^16^	EPI_ISL_15731523
SARS-CoV-2 BA.4.6 strain	Planas et al.^16^	EPI_ISL_15729633
SARS-CoV-2 BA.5.1 strain	National Reference Center for Respiratory Viruses	EPI_ISL_13017789
SARS-CoV-2 XBB.1 strain	National Reference Center for Respiratory Viruses	hCoV-19/France/PAC-HCL022171892001/2022
SARS-CoV-2 XBB.1.5 strain	Bruel et al.^22^	EPI_ISL_16353849
SARS-CoV-2 XBB.1.16.1 strain	National Reference Center for Respiratory Viruses	EPI_ISL_17383796
SARS-CoV-2 BA.2.86.1 strain	National Reference Center for Respiratory Viruses	EPI_ISL_18221650
SARS-CoV-2 EG.5.1.1 strain	National Reference Center for Respiratory Viruses	EPI_ISL_17949406
SARS-CoV-2 JN-1 strain	National Reference Center for Respiratory Viruses	EPI_ISL_18363371
SARS-CoV-1 isolate Frankfurt-1	National Reference Center for Respiratory Viruses	FFM-1
MAX Efficiency DH5α competent *E. coli*	ThermoFisher Scientific	#18258012

**Chemicals, peptides, and recombinant proteins**		

SARS-CoV-1 tri-S protein	Planchais et al.^26^	N/A
MERS-CoV tri-S protein	Planchais et al.^26^	N/A
Peroxidase-conjugated streptavidin	BD Biosciences	#554066; RRID:AB_2868972
ReadiUse ABTS Substrate Solution	AAT Bioquest	#11001
Fusion peptide (FP; 5KRSFIEDLLF NKVTLADAGFIK)	GenScript Biotech	N/A
HR2 peptide (DVDLGDISGINASVVNIQKEI DRLNEVAKNLNESLIDLQELGKYEQYIKWP)	GenScript Biotech	N/A
20-mer HR2 overlapping 5-amino acid peptides	GenScript Biotech	N/A
Polyethylenimine	Polysciences	#23966-2
Hoechst 33342	Invitrogen	#H3570
Denaturing Sample Loading Buffer	Invitrogen	#AM8547
Reducing Agent NuPAGE	Invitrogen	#NP0004
Paraformaldehyde 32% Aqueous Solution EM Grade	Electron Microscopy Sciences	#15714-S
LIVE⁄DEAD fixable aqua dead cell stain kit	Thermo Fisher Scientific	#L34957

**Critical commercial assays**		

QuickChange Site-Directed Mutagenesis kit	Agilent Technologies	#200523
Enzymatic Protein Biotinylation Kit	Sigma-Aldrich	#CS0008
Silver Stain kit	Thermo Fisher Scientific	#24612
Zymoprep Yeast Plasmid Miniprep II kit	Zymo Research	#D2004
Amino-coupling kit	Cytivia	#BR100050

**Deposited data**		

Atomic coordinates for the Cv2.3194-RBD^Wu^-Cv2.3194 Fab complex	This study	PDB: 8QH0
Atomic coordinates for the Cv2.3194-RBD^BA.4/5^-Cv2.3194 Fab complex	This study	PDB: 8QH1
Cryo-EM density map for the SARS-CoV-2 Spike-Cv2.3194 complex	This study	EMDB: EMD-18399

**Experimental models: Cell lines**		

Freestyle™ 293-F cells	Thermo Fisher Scientific	#R79007
Drosophila melanogaster S2 cell line	Thermo Fisher Scientific	#R690-07
Expi293F™ cells	Thermo Fisher Scientific	#A14527
Vero E6 cells	Cytion	#305008
U2OS-ACE2-GFP1-10 cells	Planas et al.^16^	N/A
U2OS-ACE2-GFP1-11 cells	Planas et al.^16^	N/A

**Experimental models: Organisms/strains**		

B6.Cg-Tg(K18-ACE2)2Prlmn/J mice	The Jackson Laboratory	#034860
Competent yeast cells EBY100	ATCC®	#MYA-4941

**Recombinant DNA**		

SARS-CoV-2 tri-S expression Vector Wuhan	Planchais et al.^26^	N/A
SARS-CoV-2 tri-S_6P expression Vector Wuhan	This study	N/A
SARS-CoV-2 tri-S expression Vector BA.1	Planchais et al.^26^	N/A
SARS-CoV-2 S1 expression Vector	Planchais et al.^26^	N/A
SARS-CoV-2 tri-S2 expression Vector	Planchais et al.^26^	N/A
SARS-CoV-2 tri-S2ΔHR2 expression Vector	This study	N/A
SARS-CoV-2 tri-S2ΔSH expression Vector	This study	N/A
OC43-CoV tri-S expression Vector	Planchais et al.^26^	N/A
HKU1-CoV tri-S expression Vector	Planchais et al.^26^	N/A
229E-CoV tri-S expression Vector	Planchais et al.^26^	N/A
NL63-CoV tri-S expression Vector	Planchais et al.^26^	N/A
SARS-CoV-2 RBD Wuhan in pCDNA3.1 expression Vector	Planchais et al.^26^	N/A
SARS-CoV-2 RBD_331-528_ Wuhan in pMT/BiP expression Vector	Planchais et al.^26^	N/A
SARS-CoV-2 RBD expression Vector B.1.351 (β)	Planchais et al.^26^	N/A
SARS-CoV-2 RBD expression Vector BA.1	Planchais et al.^26^	N/A
SARS-CoV-2 RBD expression Vector BA.2	Planchais et al.^26^	N/A
SARS-CoV-2 RBD expression Vector BA.2.12.1	This study	N/A
SARS-CoV-2 RBD expression Vector BA.2.3.20	This study	N/A
SARS-CoV-2 RBD expression Vector BA.3	This study	N/A
SARS-CoV-2 RBD expression Vector BA.4/5	This study	N/A
SARS-CoV-2 RBD_331-529_ expression Vector BA.4/5	This study	N/A
SARS-CoV-2 RBD expression Vector BA.4.6	This study	N/A
SARS-CoV-2 RBD expression Vector BF.7	This study	N/A
SARS-CoV-2 RBD expression Vector BA.2.75.2	This study	N/A
SARS-CoV-2 RBD expression Vector BQ.1	This study	N/A
SARS-CoV-2 RBD expression Vector BQ.1.1	This study	N/A
SARS-CoV-2 RBD expression Vector XBB.1	This study	N/A
SARS-CoV-2 RBD expression Vector XBB.1.5	This study	N/A
SARS-CoV-2 RBD expression Vector XBB.1.16	This study	N/A
SARS-CoV-2 RBD expression Vector BA.2.86	This study	N/A
SARS-CoV-2 RBD expression Vector EG.5	This study	N/A
SARS-CoV-2 RBD expression Vector JN.1	This study	N/A
ACE2 ectodomain expression Vector	Planchais et al.^26^	N/A
pcDNA™3.1/Zeo^(+)^ expression vector	Thermo Fisher Scientific	#V86020
pCoPURO plasmid	Addgene	#17533; RRID:Addgene_17533
Human antibody expression vectors	Planchais et al.^26^	N/A
Human Fab-Igγ1 expression vector	Planchais et al.^26^	N/A
Human Igα1 expression vector	Planchais et al.^26^	N/A
Human Fab-Igα1 expression vector	Planchais et al.^26^	N/A
Hulan J chain expression vector	Planchais et al.^26^	N/A
Human FcγRIA_16-292_ expression vector	This study	N/A
Human FcγRIIA_34-217_ expression vector	This study	N/A
Human FcγRIIB_43-217_ expression vector	This study	N/A
Human FcγRIIIA_17-208_ expression vector	This study	N/A
pUNO-1 SARS-CoV-2 Spike (D614)-dfur expression vector	InvivoGen	#p1-spike-df
pUNO-1 SARS-CoV-2 Spike (D614G)-dfur expression vector	InvivoGen	#p1-spike-v1-df
pUNO-1 SARS-CoV-2 Spike (Alpha)-dfur expression vector	InvivoGen	#p1-spike-v2-df
pUNO-1 SARS-CoV-2 Spike (Beta)-dfur expression vector	InvivoGen	#p1-spike-v3-df
pUNO-1 SARS-CoV-2 Spike (Epsilon)-dfur expression vector	InvivoGen	#p1-spike-v4-df
pUNO-1 SARS-CoV-2 Spike (Gamma)-dfur expression vector	InvivoGen	#p1-spike-v5-df
pUNO-1 SARS-CoV-2 Spike (Iota)-dfur expression vector	InvivoGen	#p1-spike- v6-df
pUNO-1 SARS-CoV-2 Spike (Kappa)-dfur expression vector	InvivoGen	#p1-spike- v7-df
pUNO-1 SARS-CoV-2 Spike (Delta)-dfur expression vector	InvivoGen	#p1-spike- v8-df
pUNO-1 SARS-CoV-2 Spike (Lambda)-dfur expression vector	InvivoGen	#p1-spike-v9-df
pUNO-1 SARS-CoV-2 Spike (Mu)-dfur expression vector	InvivoGen	#p1-spike-v10-df
pUNO-1 SARS-CoV-2 Spike (BA.1)-dfur expression vector	InvivoGen	#p1-spike-v11-df
pUNO-1 SARS-CoV-2 Spike (BA.2)-dfur expression vector	InvivoGen	#p1-spike-v12-df
pUNO-1 SARS-CoV-2 Spike (BA.4/5)-dfur expression vector	InvivoGen	#p1-spike-v13-df
pUNO-1 human FcγRIA expression vector	InvivoGen	#puno1-hfcgr1a
pUNO-1 human FcγRIIA expression vector	InvivoGen	#puno1-hfcgr2a
pUNO-1 human FcγRIIB expression vector	InvivoGen	#puno1-hfcgr2b
pUNO-1 human FcγRIIIAc expression vector	InvivoGen	#puno1-hfcgr3ac
SARS-CoV-2 RBD^Wu^ DMS libraries	Pruvost et al.^51^	N/A

**Software and algorithms**		

CLC Main Workbench 7 software v7.5.3	QIAGEN Aarhus A/S	https://digitalinsights.qiagen.com/products-overview/analysis-and-visualization/qiagen-clc-main-workbench/
FlowJo software (v10.7.1)	FlowJo LLC	https://www.flowjo.com/solutions/flowjo
GraphPad Prism software (v9.3.1)	GraphPad Prism Inc.	https://www.graphpad.com/
Pymol v.2.0	Molecular Graphics System	https://pymol.org/2/
AlphaFold2	Jumper et al.^52^	https://alphafold.ebi.ac.uk/
Phenix 1.19	Liebschener et al.^53^	https://phenix-online.org/download/
XDS Program Package vJun30, 2023	Kabsch, W^51^	https://xds.mr.mpg.de/
AIMLESS	Evans, P.R. and Murshudov, G.N.^54^	https://www.ccp4.ac.uk/html/aimless.html
Harmony software 4.8	Perkin Elmer	https://www.mri.cnrs.fr/fr/analyse-de-donn%C3%A9es/logiciels-et-outils/266-logiciel-propri%C3%A9taire/430-harmony-perkinelmer.html
Coot application v0.8.9	Emsley et al.^55^	https://www2.mrc-lmb.cam.ac.uk/personal/pemsley/coot/
Molprobity v4.5.2	Duke Biochemistry	http://molprobity.biochem.duke.edu/
PBDePISA	European Bioinformatics Institute	www.ebi.ac.uk/pdbe/prot_int/pistart.html
Relion v3.1	MRC Laboratory of Molecular Biology	https://relion.readthedocs.io/en/release-3.1/SPA_tutorial/Class3D.html
UCSF Chimera v1.17.3	UCSF	https://www.cgl.ucsf.edu/chimera/download.html
CryoSPARC v4.4.1	Structura Biotechnology Inc.	https://cryosparc.com/download
EPU automated image acquisition software	Thermo Fisher Scientific	N/A
MotionCor2	UCSF	http://msg.ucsf.edu/software
SynergyFinder web application (v3.0)	SynergyFinder	https://synergyfinder.fimm.fi
BIAevaluation version 4.1.1 Software	Cytivia	N/A

**Other**		

Ni Sepharose Excel Resin	Cytiva	#17371202
Protein G sepharose 4 fast flow beads	GE Healthcare	#17061805
Peptide M-coupled agarose beads	Invivogen	#gel-pdm-5
Slide-A-Lyzer® dialysis cassettes	Thermo Fisher Scientific	#10065983
NanoDrop2000 instrument	Thermo Fisher Scientific	N/A
NuPAGE 3-8% Tris-acetate gels	Thermo Fisher Scientific	#EA03785BOX
Nitrocellulose blotting membrane	Cytivia	#10600008
Miniblot apparatus	Immunetics	N/A
Strep-Tactin Superflow high-capacity column	IBA Life Sciences	#2-1238-001
Superose6 10/300 column	Cytivia	N/A
Superdex75 column	Cytivia	N/A
Superdex 200 column	Cytiva	N/A
High binding 96-well ELISA plates	Corning	#9018
μClear 96-well plate	Greiner Bio-One	# 655094
HydroSpeed™ microplate	Tecan Männedorf	INSTHS-02
Cytoflex flow cytometer	Beckman Coulter	N/A
iBright™ FL1500 Imaging System	Thermo Fisher Scientific	N/A
Opera Phenix high-content confocal microscope	Perkin Elmer	N/A
Biacore 2000	Cytivia	N/A
CM5 sensor chips	Cytivia	#29104988
Vitrobot Mk IV	Thermo Fisher Scientific	N/A
Titan Krios transmission electron microscope	Thermo Fisher Scientific	N/A
Gatan K3 direct electron detector	Gatan, inc.	N/A
Sunrise™ microplate absorbance reader	Tecan Männedorf	N/A
BD FACS Aria III sorter	Becton Dickinson	N/A
Illumina MiSeq device	Illumina	N/A

### Resource availability

#### Lead contact

Requests for resources and reagents should be directed to and will be fulfilled by the lead contact, Hugo Mouquet (hmouquet@pasteur.fr).

#### Materials availability

Request for reagents will be made available by the lead contact with a Material Transfer Agreement.

#### Data and code availability

• Atomic coordinates for the Cv2.3194-RBD^Wu^ and Cv2.3194-RBD^BA.4/5^ complexes were deposited in the Protein Data Bank, and the cryo-EM density map for the SARS-CoV-2 Spike-Cv2.3194 complex was deposited in the Electron Microscopy Data Bank, and are publicly available as of the date of publication. Accession numbers are listed in the key resources table. All data reported in this paper will be shared by the lead contact upon request.
• This paper does not report original code.
• Any additional information required to reanalyse the data reported in this paper is available from the lead contact upon request.

### Method details

#### Viruses and recombinant viral proteins

The SARS-CoV-2 viral stocks for the following strains used in the *in vitro* S-Fuse neutralization assay were prepared and titrated in the Viral and Immunity Unit (Institut Pasteur) as previously described^16^^,^^17^: reference D614G strain (hCoV-19/France/GE1973/2020, GISAID ID: EPI_ISL_414631; National Reference Center for Respiratory Viruses (Institut Pasteur)), BA.2.75.2 (GISAID ID: EPI_ISL_15731524), BQ.1.1 (GISAID ID: EPI_ISL_15731523), BA.4.6 (GISAID ID: EPI_ISL_15729633), XBB.1 (hCoV-19/France/PAC-HCL022171892001/2022)), XBB.1.5 (GISAID ID: EPI_ISL_16353849), XBB.1.16.1 (GISAID ID: EPI_ISL_17383796), BA.2.86.1 (GISAID ID: EPI_ISL_18221650), EG.5.1.1 (GISAID ID: EPI_ISL_17949406) and JN.1 (GISAID ID: EPI_ISL_18363371).^16^^,^^17^ The Beta strain (β, B.1.351; hcoV-19/France/IDF-IPP00078/2021) and BA.5.1 strain (GISAID id: EPI_ISL_13017789) used for mouse experiments was supplied by the National Reference Centre for Respiratory Viruses (Institut Pasteur, France). All work with infectious virus was performed in biosafety level 3 containment laboratories at Institut Pasteur.

Trimeric SARS-CoV-2, SARS-CoV-1, MERS-CoV, OC43-CoV, HKU1-CoV, 229E-CoV, NL63-CoV, BA.1 ectodomains (tri-S); SARS-CoV-2 tri-S2, tri-S2ΔHR2 and tri-S2ΔSH proteins, S1 subunit, and human angiotensin-converting enzyme 2 (ACE2) ectodomain, and RBD proteins (Wuhan to BA.2) cloned into pcDNA3.1/Zeo(+) vector were previously described.^26^ For the additional mutated SARS-CoV-2 RBD proteins (BA.4/5 to JN.1), mutations (Figure S2A) were introduced using the QuickChange Site-Directed Mutagenesis kit (Agilent Technologies) following the manufacturer’s instructions or obtained with synthetic DNA fragments (GeneArt, Thermo Fisher Scientific). Recombinant proteins were produced by transient transfection of exponentially growing Freestyle 293-F suspension cells (Thermo Fisher Scientific) using polyethylenimine (PEI) precipitation method, purified from culture supernatants by high-performance chromatography using the Ni Sepharose® Excel Resin according to manufacturer’s instructions (GE Healthcare), dialyzed against PBS using Slide-A-Lyzer® dialysis cassettes (Thermo Fisher Scientific), quantified using NanoDrop 2000 instrument (Thermo Fisher Scientific), and controlled for purity by SDS-PAGE using NuPAGE 3-8% Tris-acetate gels (Life Technologies) as previously described.^26^ AviTagged tri-S and RBD proteins were biotinylated using the Enzymatic Protein Biotinylation Kit (Sigma-Aldrich).

For crystallographic experiments, the SARS-CoV-2 RBD (residues 331-528) cloned into a modified pMT/BiP plasmid (Invitrogen; hereafter termed pT350), was produced by stable transfection of *Drosophila* S2 cells with the pCoPuro plasmid for puromycin selection and purified by affinity chromatography using StrepTactin beads and size-exclusion chromatography (SEC) on a Superdex75 (Cytiva) column as previously described.^26^ The BA4/5 RBD (residues 331-529, Wuhan numbering) cloned into pcDNA3.1/Zeo(+) was produced and purified as described above. For Cryo-EM experiments, a tagged, stabilized and trimerized recombinant version of the SARS-CoV-2 Spike (S) ectodomain (residues 1-1208) designated S_6P,^56^ was produced by transient transfection of Expi293F™ cells (Thermo Fischer and purified by affinity chromatography using StrepTactin beads and size-exclusion chromatography (SEC) on a Superose6 10/300 column (Cytiva) as previously described.^26^

#### Recombinant antibodies, Fabs and soluble Fcγ receptors

Human anti-SARS-CoV2 Cv2.1169, Cv2.3132, Cv2.3194,^26^ S309^53^ and LY-CoV1404^57^ and negative control (mGO53^58^) IgG1 antibodies were produced by transient co-transfection of Freestyle™ 293-F suspension cells (Thermo Fisher Scientific) and purified by affinity chromatography using Protein G Sepharose® 4 Fast Flow (GE Healthcare) as previously described.^26^ Cv2.3194 IgH was also cloned into human Igα1 and Fab-Igγ1-expressing vectors, and recombinant Cv2.3194 IgA1 antibodies and Fabs were produced and purified using peptide M-coupled agarose beads (Invivogen) and Ni Sepharose® Excel Resin (GE Healthcare), respectively as previously described.^26^ Monomeric and dimeric Cv2.3194 IgA1 antibodies were separated by size-exclusion chromatography (SEC) using a Superose 6 Increase 10/300 column (Cytiva) previously described.^26^ The quality/purity of the different purified fractions was evaluated by SDS-PAGE using 3–8% Tris–Acetate gels (Life Technologies) under non-reducing conditions followed by silver staining (Silver Stain kit, Thermo Scientific). Purified antibodies and Fabs were dialyzed against PBS.

Codon-optimized nucleotide fragments encoding FcγRIA (P12314, 16-292 residues), FcγRIIA (P12318, 34-217 residues), FcγRIIB (P31994, 43-217 residues) and FcγRIIIA (P08637, 17-208 residues) ectodomains preceded by the human IgK leader peptide and followed C-terminal tags (Hisx8-tag and AviTag) were synthesized and cloned into pcDNA3.1/Zeo(+) expression vector (Thermo Fisher Scientific). Soluble recombinant Fcγ receptors were produced by transient transfection Freestyle 293-F cells and purified using Ni Sepharose® Excel Resin as described above.

#### ELISAs

ELISAs were performed as previously described.^26^ Briefly, high-binding 96-well ELISA plates (Costar, Corning) were coated overnight with 250 ng/well of purified recombinant Coronavirus proteins. After washings with 0.05% Tween 20-PBS (washing buffer), plates were blocked 2 h with 2% BSA, 1 mM EDTA, 0.05% Tween 20-PBS (Blocking buffer), washed, and incubated with serially diluted purified IgG mAbs in PBS. Recombinant IgG1 antibodies were tested at 10 μg/ml, and 7 consecutive 1:4 dilutions in PBS. To evaluate the binding of recombinant IgG1 mAbs to human FcγRs (FcγRIA, FcγRIIA, FcγRIIB and FcγRIIIA), ELISAs plates (Costar, Corning) were coated were coated overnight with 250 ng/well of purified recombinant FcγRs. After washings with 0.05% Tween 20-PBS (washing buffer), plates were blocked 2 h with 2% BSA, 1 mM EDTA, 0.05% Tween 20-PBS (Blocking buffer), washed, and incubated with serially diluted purified IgG mAbs alone or in presence of biotinylated RBD at 0.5 μg/ml in PBS. Recombinant IgG1 antibodies were tested at 10 μg/ml, and 7 consecutive 1:3 dilutions in PBS. After washings, the plates were revealed by incubation for 1 h with goat HRP-conjugated anti-human IgG (Jackson ImmunoReseach, 0.8 μg/ml final) or for 30 min with streptavidin HRP-conjugated (BD Biosciences) and by adding 100 μl of HRP chromogenic substrate (ABTS solution, Euromedex) after washing steps. Optical densities were measured at 405nm (OD_405nm_), and background values given by incubation of PBS alone in coated wells were subtracted. Experiments were performed using HydroSpeed™ microplate washer and Sunrise™ microplate absorbance reader (Tecan Männedorf, Switzerland). For peptide-ELISA, binding of SARS-CoV-2 and control IgG antibodies to the fusion peptide (FP; KRSFIEDLLFNKVTLADAGFIK), HR2 peptide (DVDLGDISGINASVVNIQKEIDRLNEVAKNLNESLIDLQELGKYEQYIKWP), and 20-mer HR2 overlapping 5-amino acid peptides (n=8) (GenScript Biotech, 500 ng/well) was tested using the same procedure as previously described.^26^ For the competition experiments of tri-S-binding to ACE2, ELISA plates (Costar, Corning) were coated overnight with 250 ng/well of purified ACE2 ectodomain. After washings, plates were blocked 2 h with Blocking buffer, PBST-washed, and incubated with recombinant IgG1 mAbs at 10 μg/ml and 7 consecutive 1:2 dilutions in presence of biotinylated tri-S protein at 1 μg/ml in PBS. After washings, the plates were revealed by incubation for 30 min with streptavidin HRP-conjugated (BD Biosciences) as described above.

#### Flow cytometry binding assay

To evaluate spike cross-reactivity and binding to human FcγRs, Freestyle™ 293-F were transfected with pUNO1-Spike-dfur (Spike and SpikeV1 to V13 plasmids, Invivogen) or pUNO1 bearing the human FcγR genes (hfcgr1a, hfcgr2a, hfcgr2b and hfcgr3a plasmids, Invivogen) expression vectors (1.2 μg plasmid DNA *per* 10^6^ cells) using PEI-precipitation method. Forty-eight hours post-transfection, 0.5x10^6^ transfected and non-transfected control cells were incubated with IgG antibodies for 30 min at 4°C. Antibodies were tested at 1 μg/ml for spike cross-reactivity experiments and concentrations are ranging from 0.08 to 5 μg/ml for FcγR binding assays. After washings, cells were incubated 20 min at 4°C with AF647-conjugated goat anti-human IgG antibodies (1:1000 dilution; Thermo Fisher Scientific) and LIVE/DEAD Fixable Viability dye Aqua (1:1000 dilution; Thermo Fisher Scientific), washed and resuspended in PBS-Paraformaldehyde 1% (Electron Microscopy Sciences). Data were acquired using a CytoFLEX flow cytometer (Beckman Coulter) and analyzed using FlowJo software (v10.7.1; FlowJo LLC). Antibodies were tested in duplicate.

#### Infrared immunoblotting

Recombinant tri-S proteins (Wuhan tri-S, BA.1 tri-S and tri-S2) were heat-denatured at 100°C for 3 min in loading buffer (Invitrogen) containing 1X sample reducing agent (Invitrogen), and 0.5% SDS. Nitrocellulose membranes were hydrated with PBS and inserted into a Miniblot apparatus (Immunetics). Native and denatured tri-S proteins (10 μg total) were incubated on membranes for 2 h in PBS, and saturated in 5% dry milk-PBS-0.05% Tween 20 (PBST) overnight at 4°C. Membranes were rotated 90° clockwise, inserted into a Miniblot apparatus and then incubated for 2 h with human IgG antibodies (at a concentration ranging from 0.08 to 10 μg/ml) and a mouse anti-Hisx6 antibody (1 μg/ml, BD Biosciences) in 5% dry milk-PBST in each channel. After PBST washings, membranes were incubated for 1 h with 1:25,000-diluted Alexa Fluor 680-conjugated donkey anti-human IgG (Jackson ImmunoResearch) and 1:25,000-diluted IR Dye® 800CW-conjugated goat anti-mouse IgG (LI-COR Biosciences) in 5% dry milk-PBST. Finally, membranes were washed and scanned using the iBright™ FL1500 Imaging System (Invitrogen, Thermo Fisher Scientific).

#### Surface plasmon resonance

Surface plasmon resonance (SPR)-based technology (Biacore 2000, Biacore, Cytiva Uppsala, Sweden) was used to assess the kinetics of interaction of Cv2.3194 and Cv2.3132 with the trimeric recombinant SARS-CoV-2 protein (tri-S). Purified Wuhan tri-S (S_6P) was covalently immobilized to CM5 sensor chips (Biacore) using amino-coupling kit (Biacore Cytiva) as previously described.^26^ All interactions were performed at temperature of 25°C. The flow rate of the HBS-EP buffer (10 mM HEPES pH 7.2; 150 mM NaCl; 3 mM EDTA, and 0.005 % Tween 20) during all real-time interaction measurements was set at 30 μl/min. For assessment of binding kinetics, purified IgG antibodies were serially diluted (two-fold step) in HBS-EP in the range of 25 – 0.39 nM. The association and dissociation phases of the interaction process were monitored for 4 or 5 minutes. The binding of the proteins to reference channel containing carboxymethylated dextran only, was used as a negative control and was always subtracted from the binding to spike protein during data processing. The sensor chip surfaces were regenerated by a brief (30 s) exposure to 3M solution of KSCN (Sigma-Aldrich). The evaluation of the kinetic parameters of the studied interactions were performed by global analyses using BIAevaluation version 4.1.1 Software (Biacore). The dual binding experiments were performed by consecutive injections of 80 nM of Cv2.3132 followed by 80 nM of Cv2.3194, without regeneration and vice versa. The association times were monitored for 4 min and dissociation was followed for 5 min minimum.

#### SARS-CoV-2 S-Fuse neutralization assay

S-Fuse cells (1:1 mix of U2OS-ACE2-GFP1-10 and U2OS-ACE2-GFP11) were prepared and plated at a density of 2 × 10^4^ *per* well in a μClear 96-well plate (Greiner Bio-One) as previously described.^16^ SARS-CoV-2 VOC viruses (MOI 0.1) were incubated with recombinant IgG1 mAbs at 100 μg/ml, and 17 consecutive 1:2 dilutions in culture medium for 30 min at room temperature and added to S-Fuse cells. The cells were fixed, 18 h later, in 4% paraformaldehyde, washed and stained with Hoechst stain (dilution 1:10,000; Invitrogen). Images were acquired with an Opera Phenix high-content confocal microscope (Perkin Elmer). The area displaying GFP expression, and the number of nuclei were quantified with Harmony software 4.8 (Perkin Elmer). The percentage neutralization was calculated from the number of syncytia as follows: 100 × (1 – (value with IgG – value in “non-infected”) / (value in “no IgG” – value in “non-infected”)). IC_50_ values were calculated using Prism software (v.9.3.1, GraphPad Prism Inc.) by fitting replicate values using the four-parameters dose–response model (variable slope). For evaluating the synergistic potential of combined antibodies (Cv2.3194+Cv2.3132), S-Fuse neutralization experiments were performed using BA.4.6 and XBB.1.5 strains as described above but using mixed antibodies at different concentration ratio in comparison to the single antibodies alone (0). For BA.4.6, Cv2.3132 at 150 μg/ml and 10 three-fold dilutions was mixed with Cv2.3134 at 15 μg/ml and 10 three-fold dilutions. For XBB.1.5, Cv2.3132 at 150 μg/ml and 10 three-fold dilutions was mixed with Cv2.3134 at 150 μg/ml and 10 two-fold dilutions. The % neutralization was calculated for each combination. Two independent experiments were performed, and mean values were calculated. The quantification of the synergistic effect of the two antibodies in the cocktail was calculated using the SynergyFinder web application (v3.0) (https://synergyfinder.fimm.fi),^52^ with the corrected Zero interaction potency (ZIP) model. A global synergy score < −10 indicates antagonism, ranging from −10 to 10 indicates an additive effect, and < 10 indicates synergy.

#### SARS-CoV-1 neutralization assay

Plaque-reduction neutralization test (PRNT) experiments were conducted under strict BSL3^+^ conditions. Hundred PFU of SARS-CoV-1 isolate Frankfurt-1 (FFM-1) mixed with serially diluted IgG antibodies in DMEM were incubated for 30 min at 37°C. Mixtures (100 μl) were then added to Vero E6 cells pre-seeded in 24-well tissue culture plates (2.10^5^ cells *per* well) and incubated at 37°C and 5% CO_2_ under agitation. After 1 h adsorption, a liquid overlay comprising 1% carboxymethylcellulose diluted in DMEM without FBS supplemented with 0.1% Penicillin/Streptomycin was added to each well and plates were incubated for 72 h. The liquid overlay was removed, and cells were fixed and stained with 30% crystal violet, 20% ethanol and 10% formaldehyde for 10 min. Plates were washed and plaques were enumerated and compared to controls. IC_50_ values were calculated using Prism software (v.9.3.1; GraphPad Prism Inc.) by fitting triplicate values using the four-parameter dose–response model (variable slope).

#### SARS-CoV-2 infection and treatment in K18-hACE2 mice

B6.Cg-Tg(K18-ACE2)2Prlmn/J mice (stock #034860) were imported from The Jackson Laboratory (Bar Harbor, ME, USA), and bred at the Institut Pasteur under strict SPF conditions. Infection studies were performed on 6 to 16 wk-old male and female mice, in animal biosafety level 3 (BSL-3) facilities at the Institut Pasteur, in Paris. All animals were handled in strict accordance with good animal practice. Animal work was approved by the Animal Experimentation Ethics Committee (CETEA 89) of the Institut Pasteur (project dap 200008 and 200023) and authorized by the French legislation (under project 24613) in compliance with the European Communities Council Directives (2010/63/UE, French Law 2013–118, February 6, 2013) and according to the regulations of Pasteur Institute Animal Care Committees before experiments were initiated. Anesthetized (ketamine/xylazine) mice were inoculated intranasally (i.n.) with 1 x10^4^ PFU of SARS-CoV-2 Virus Beta 3.1.351 (20 μl/nostril). Six hours pre- or post-inoculation, mice received an intraperitoneal (i.p.) injection of 10 mg/kg of Cv2.3132 or mGO53 control IgG antibody. Clinical signs of disease (ruffled fur, hunched posture, reduced mobility, and breathing difficulties) and weight loss were monitored for 16 days. Mice were euthanized when they reached pre-defined end-point criteria. Sera were extracted from blood collected by puncture of the retromandibular vein. Mouse survivals were compared across groups using a Kaplan-Meier analysis and Log-rank Mantel-Cox test (GraphPad Prism, v8.2, GraphPad Prism Inc.).

#### Yeast display-based deep mutational scanning

Epitope mapping experiments with Cv2.1169 and Cv2.3194 were performed as previously described.^50^ Briefly, four libraries coding for the SARS-CoV2 RBD (original strain) with single amino acid mutations were constructed using splicing by overlap extension PCR (SOE-PCR) and degenerate NNK primers covering amino acid 1 to 211 (residues numbering referring to PDB: 6M0J). Library 1 corresponds to amino acids 1 to 64, library 2: amino acids 65 to 128, library 3: amino acids 129 to 192 and library 4: amino acids 193 to 211. Induced yeast cell libraries were induced, washed with 1ml of ice-cold PBSF buffer (phosphate-buffered saline (PBS), bovine serum albumin (BSA) 0.1%) and incubated with 1 nM Cv2.3194 Fab / 5 nM Dylight^650^-conjugated Sotrovimab in PBSF solution for 2h at 20°C under agitation at 1000 rpm. After PBSF washing, cells were incubated with anti-His antibody (Invitrogen #MA1-21315, Dylight550 conjugate, 1:100 dilution) for 15 min on ice before analysis on a BD FACS Aria III cytometer. Gates were defined to sort the cells with decreased binding for Cv2.1169 or Cv2.3194 Fab without alteration for Sotrovimab binding (10% of total cells). After sorting, cells were cultured for two days in SD-CAA medium at 30°C. Extraction of plasmids, preparation of DNA libraries for Illumina NGS sequencing and data analysis were performed as previously described.^50^

#### Crystallization and structural determination

SARS-CoV-2 and BA.4/BA.5 RBD proteins (RBD^Wu^ and RBD^BA.4/5^, respectively) were incubated with CV2.3194 Fab (RBD:Fab molar ratio of 1.5:1) at 4°C overnight. Each binding reaction was loaded onto a Superdex200 column (Cytiva) equilibrated in 10 mM Tris-HCl (pH 8.0), 100 mM NaCl. The fractions corresponding to the complexes were analyzed by SDS-PAGE, pooled, concentrated to 9-10 mg/ml and used in crystallization trials at 18°C using the sitting-drop vapor diffusion method following established protocols.^55^ The CV2.3194-RBD^Wu^ complex crystalized in 20% PEG 3350, 0.2 M lithium citrate, while crystals for CV2.3194-RBD^BA.4/5^ were obtained with 20% PEG 3350, 0.2 M sodium malonate. Crystals were flash-frozen by immersion into a cryo-protectant containing the crystallization solution supplemented with 30% (v/v) glycerol, followed by rapid transfer into liquid nitrogen. Data collection was carried out at the European Synchrotron Radiation Facility (ESRF; Grenoble, France). Data were processed, scaled and reduced with XDS^51^ and AIMLESS.^54^ The structures were determined by molecular replacement using Phaser from the PHENIX suite^59^ with search ensembles obtained from the PBDs 7QF0 (Wuhan RBD), 7ZXU (BA4/5 RBD), and a CV2.3194 model generated by AlphaFold2.^49^ The final models were built by combining real space model building in Coot^60^ with reciprocal space refinement with phenix.refine. The final model was validated with Molprobity.^61^ Epitope and paratope residues and their interactions were identified using PISA at the European Bioinformatics Institute (www.ebi.ac.uk/pdbe/prot_int/pistart.html).^62^ Superpositions and figures were rendered using Pymol^63^ and UCSF Chimera.^64^

#### Cryo-EM sample preparation, imaging and image processing

The SARS-CoV-2 Spike (S_6P) was incubated with the Cv2.3194 Fab at a 1:6 (S_6P:Fab) ratio and a final trimer concentration of 0.8 μM for 1h at room temperature. Sample aliquots (3 μl) were applied to freshly glow discharged R 1.2/1.3 Quantifoil grids prior to plunge freezing using a Vitrobot Mk IV (Thermo Fischer Scientific) at 8°C and 100% humidity (blot 4s, blot force 0). Data for the complex were acquired on a Titan Krios transmission electron microscope (Thermo Fischer Scientific) operating at 300 kV, using the EPU automated image acquisition software (Thermo Fisher Scientific). Movies were collected on a Gatan K3 direct electron detector operating in Counted mode at a nominal magnification of 105,000x (0.86 Å/pixel) using defocus range of -1.0 μm to -3.0 μm. Movies were collected over a 1.6 s exposure and a total dose of ∼50 e^-^/Å^2^. All movies were motion-corrected and dose-weighted with MotionCorr2^65^ and the aligned micrographs were used to estimate the defocus values with patchCTF within cryosparc.^66^ CryoSPARC blob picker was used for automated particle picking and the resulting particles used to obtain initial 2D references, which were then used to auto-pick the micrographs. An initial 3D model was obtained in cryosparc and used to perform a 3D classification without imposing any symmetry in Relion.^67^ The best class was selected and subjected to 3D, non-uniform refinement in cryosparc.^68^
