# Supplementary material for: Broad sarbecovirus neutralization by combined memory B cell antibodies to ancestral SARS-CoV-2
Source: iScience. 2024 Jun 22;27(7):110354. doi: 10.1016/j.isci.2024.110354 (PMC11277385; doi:10.1016/j.isci.2024.110354)
Supplement: Document S1. Figures S1–S6 and Tables S1–S4 [file mmc1.pdf]

iScience, Volume *27*

Supplemental information

Broad sarbecovirus neutralization by combined memory B cell antibodies to ancestral SARS-CoV-2

Cyril Planchais, Ignacio Fernández, Benjamin Chalopin, Timothée Bruel, Pierre Rosenbaum, Maxime Beretta, Jordan D. Dimitrov, Laurine Conquet, Flora Donati, Matthieu Prot, Françoise Porrot, Delphine Planas, Isabelle Staropoli, Florence Guivel-Benhassine, Eduard Baquero, Sylvie van der Werf, Ahmed Haouz, Etienne Simon-Lorière, Xavier Montagutelli, Bernard Maillère, Félix A. Rey, Pablo Guardado-Calvo, Hervé Nozach, Olivier Schwartz, and Hugo Mouquet

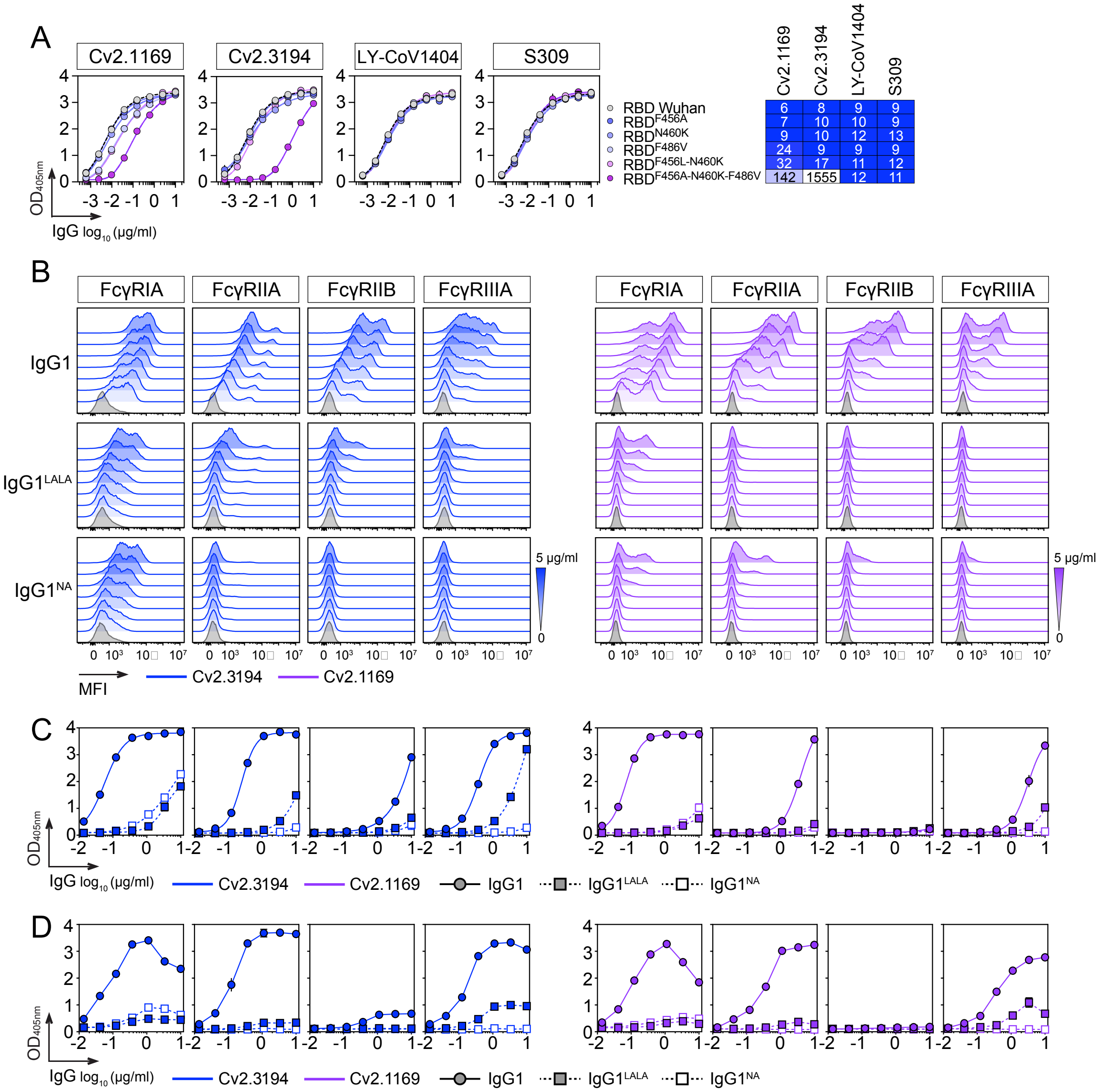

Figure S1. Binding profiles of Cv2.1169 and Cv2.3194 to mutated RBD proteins and Fcγ receptors, related to Figures 1 and 2.

(**A**) Representative ELISA graphs showing the binding of Cv2.1169, Cv2.3194 and control antibodies (LY-CoV1404 and S309) antibodies to purified wildtype and mutated SARS-CoV-2 RBD proteins (left). Heatmap comparing the binding EC_{50} values (ng/ml) of the antibodies to RBD proteins (right).

(**B**) Representative flow cytometric histograms comparing the binding of Cv2.1169 (blue), Cv2.3194 (purple) and respective Fc mutant antibodies (N297A [$IgG1^{NA}$] and L234A/L235A [$IgG1^{LALA}$], dotted lines) to human Fc γRIA, FcγRIIA, FcγRIIB and FcγRIIIA. Antibody concentrations are ranging from 0.08 to 5 µg/ml. Grey histograms indicate no antibodies.

(**C**) ELISA graphs showing the binding of Cv2.1169, Cv2.3194 and respective $IgG1^{NA}$/$IgG1^{LALA}$ mutant antibodies to purified soluble human FcγRIA, FcγRIIA, FcγRIIB and FcγRIIIA ectodomains. Error bars indicate the SD of duplicate values.

(**D**) Same as in (C) but with biotinylated RBD^{Wu}-antibody immune complexes.

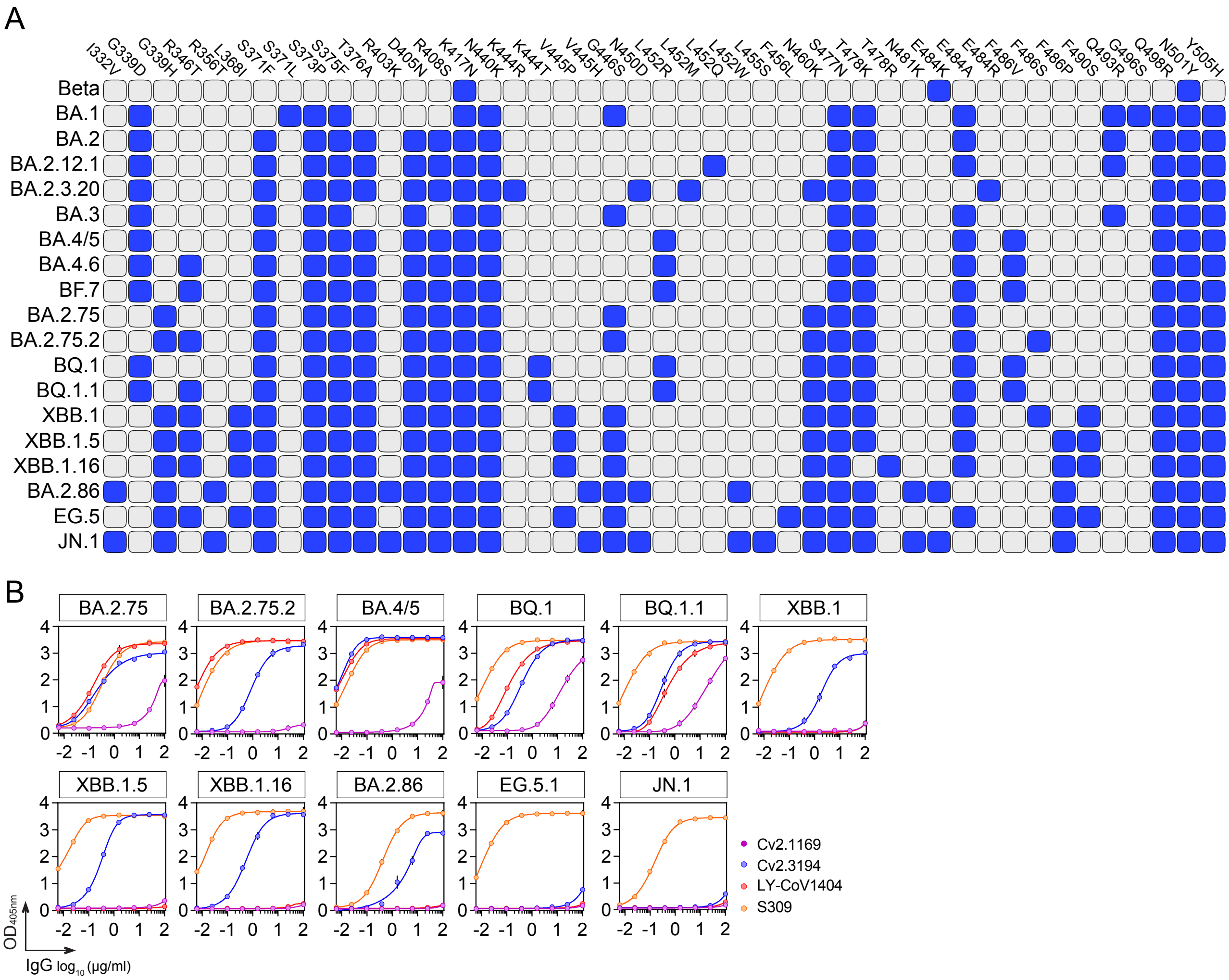

Figure S2. RBD mutant proteins and antibody binding, related to Figures 1 and 2.
(**A**) Diagram showing the amino acid substitutions present in the diverse RBD proteins used for binding analyses depicted in Figures 1B, 2A and 2B.
(**B**) Representative graph showing the ELISA binding curves of Cv2.1169, Cv2.3194 and control antibodies (LY-CoV1404 and S309) at high concentrations against selected purified RBD proteins of SARS-CoV-2 VOCs. Error bars indicate the SD of duplicate values.

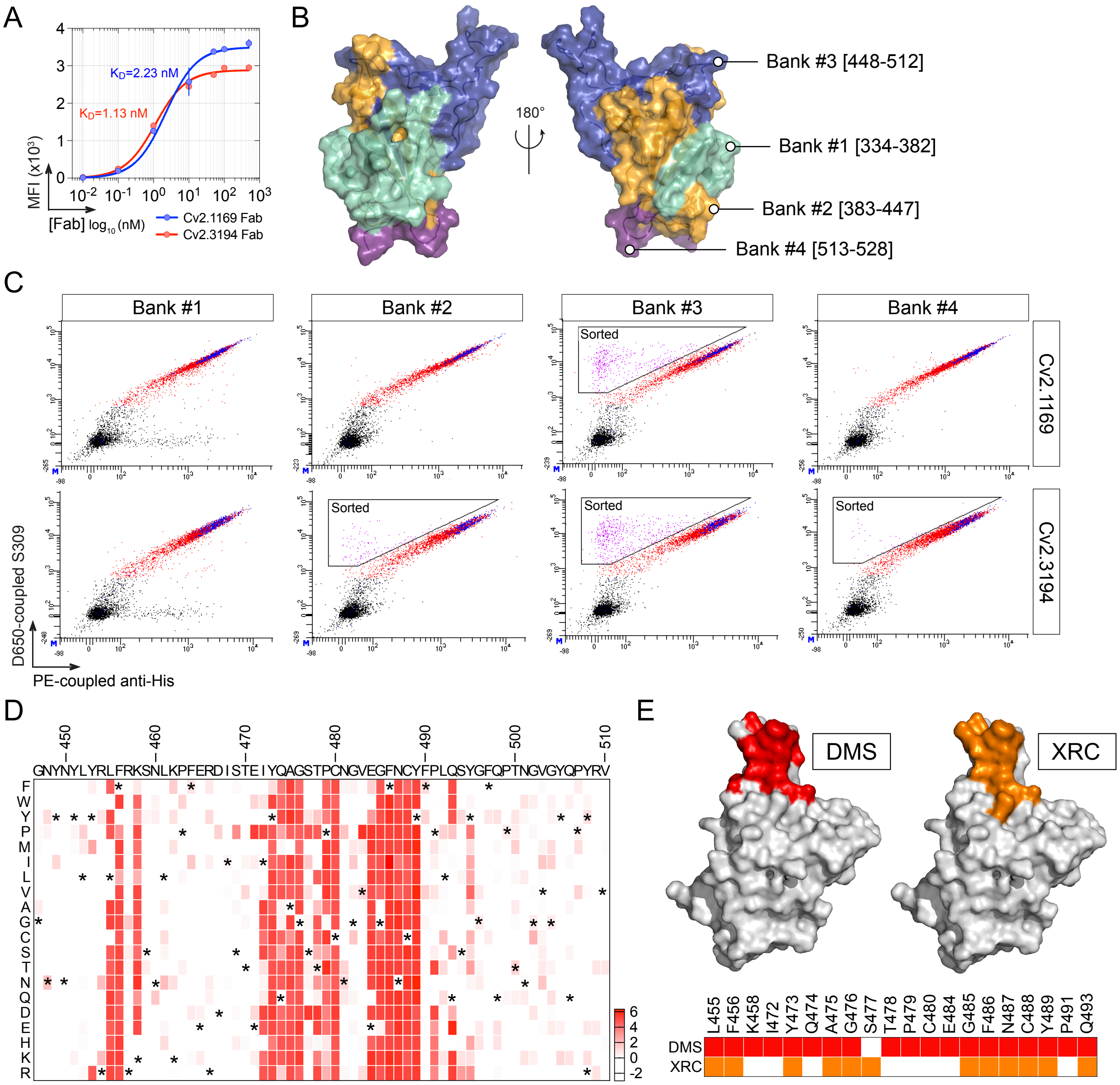

Figure S3. Mapping of Cv2.1169 and Cv2.3194 epitopes by deep mutational scanning, related to Figure 3.
(**A**) Graph showing the binding curves of Cv2.1169 and Cv2.3194 Fabs to yeast-expressed SARS-CoV-2 RBD as measured by flow cytometry. Means ± SD of duplicate values are shown. MFI, mean fluorescence intensity.
(**B**) Surface diagrams depicting the regions corresponding to the mutant libraries (banks) used for the DMS experiments on the RBD structure (PDB ID: 7QEZ).
(**C**) Flow cytometer plots showing the binding of Cv2.1169 and Cv2.3194 Fabs to yeast-expressed SARS-CoV-2 RBD mutants from the 4 libraries and revealed using an anti-HIS-tag antibody probe (X axis). The expression level of the RBD proteins was controlled using fluorescently labeled Sotrovimab.
(**D**) NGS-based heatmap showing the enrichment scores of RBD single mutants after functional sorting by FACS using Cv2.1169 Fab as bait. Enrichment score is a log2 function of the frequency fold-change between sorted and unsorted RBD yeast populations for a given amino acid substitution. The corresponding table is colored in red for enriched mutations. The index is set as the number of substitutions with an enrichment score higher than 2. Asterisks indicate the original amino acid residues.
(**E**) Surface diagrams comparing the binding fingerprints of Cv2.1169 Fab on the SARS-CoV-2 RBD obtained by deep mutational scanning (DMS; red) and X-ray crystallography (orange) (top). Table comparing the Cv2.1169 Fab-RBD interacting residues identified by DMS and X-ray crystallography (bottom).

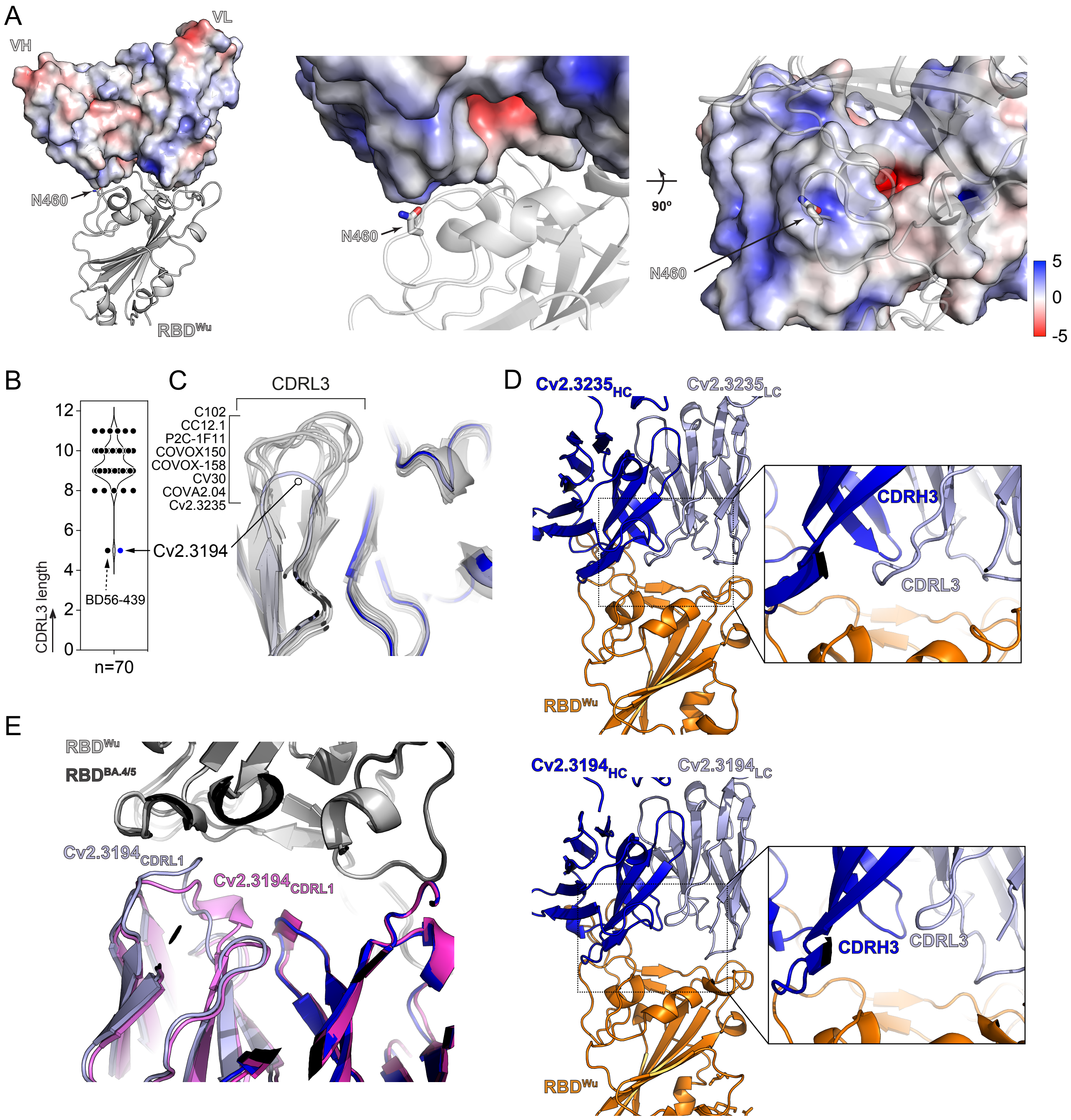

Figure S4. Structural characterization of Cv2.3194-SARS-CoV-2 RBD complex, related to Figure 4.

(**A**) Structure of the Cv2.3194-RBD^{Wu} complex with the RBD shown in gray cartoon and the V_H-V_L domains of the antibody in surface colored according to the electrostatic potential. The position of N460 is highlighted. The central panel is a magnification of the area around N460.

(**B**) Dot plot showing the CDR_L3 length of Cv2.3235, Cv2.3194 and V_H3-53-encoded class 1 RBD antibodies[14].

(**C**) Structural superposition of the CDR_L3 from Cv2.3194 (5 residues long; blue-colored) and other V_H3-53 class 1 RDB antibodies: C102 (PDB ID: 7K8M), CC12.1 (PDB ID: 6XC2), P2C-1F11 (PDB ID: 7CDI), COVOX-150 (PDB ID: 7BEI), COVOX-158 (PDB ID: 7BEJ), CV30 (PDB ID: 6XE1), COVA2-04 (PDB ID: 7JMO) and Cv2.3235 (PDB ID: 7QF0).

(**D**) Structural comparison of Cv2.3235-RBD^{Wu} (top) and Cv2.3194-RBD^{Wu} (bottom) complexes. Enlarged insets on the right show the positioning of the CDR_H3 and CDR_L1 loops in the paratope of Cv2.3235 and Cv2.3194.

(**E**) Structural superposition of the Cv2.3194-RBD^{Wu} and Cv2.3194-RBD$^{BA.4/5}$ complexes, with a close-up at the interface highlighting the different conformation of the CDR_L1 when Cv2.3194 is in complex with RBD^{Wu} (light blue) or RBD$^{BA.4/5}$ (light purple).

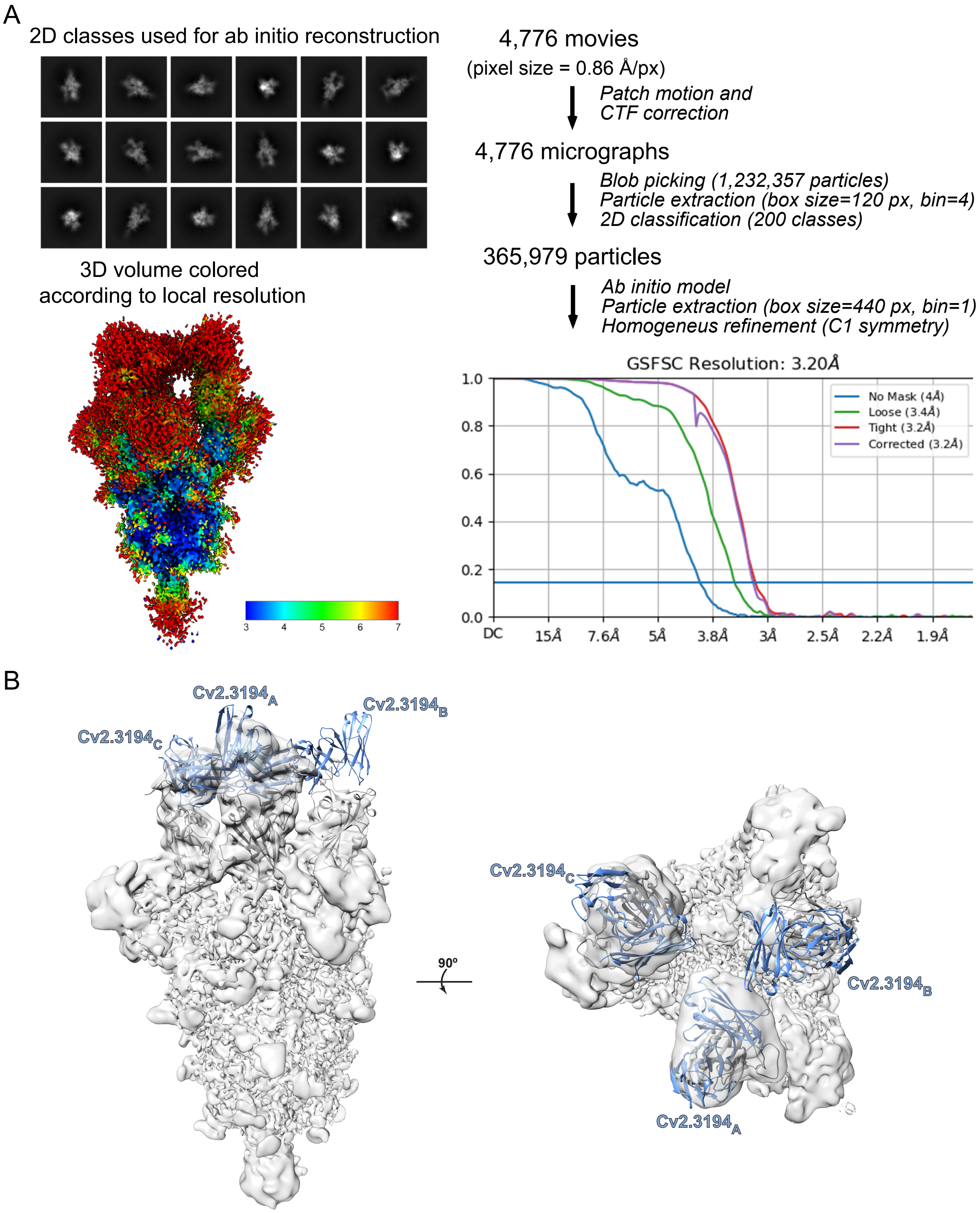

Figure S5. Cryo-EM data collection and processing of the Cv2.3194-S_6P complex, related to Figure 4.
(**A**) A micrograph with particles, selected two-dimensional class averages, a local resolution graphic, and a scheme with the steps followed to process the collected data (along with GSFSC resolution plot) are shown for the Cv2.3194-S_6P complex.
(**B**) Fitting of the Cv2.3194-RBD^{Wu} crystal structure into the cryo-EM map of the SARS-CoV-2 spike complexed with the Fab. The map has been filtered by resolution and only the variable domains of the Fab are represented due to the absence of observed densities for the constant regions.

A Peptide #

1 DVDLGDISGINASVVNIQKE
2 DISGINASVVNIQKEIDRLN
3 NASVVNIQKEIDRLNEVAKN
4 NIQKEIDRLNEVAKNLNESL
5 IDRLNEVAKNLNESLIDLQE
6 EVAKNLNESLIDLQELGKYE
7 LNESLIDLQELGKYEQYIKW
8 IDLQELGKYEQYIKWP

B

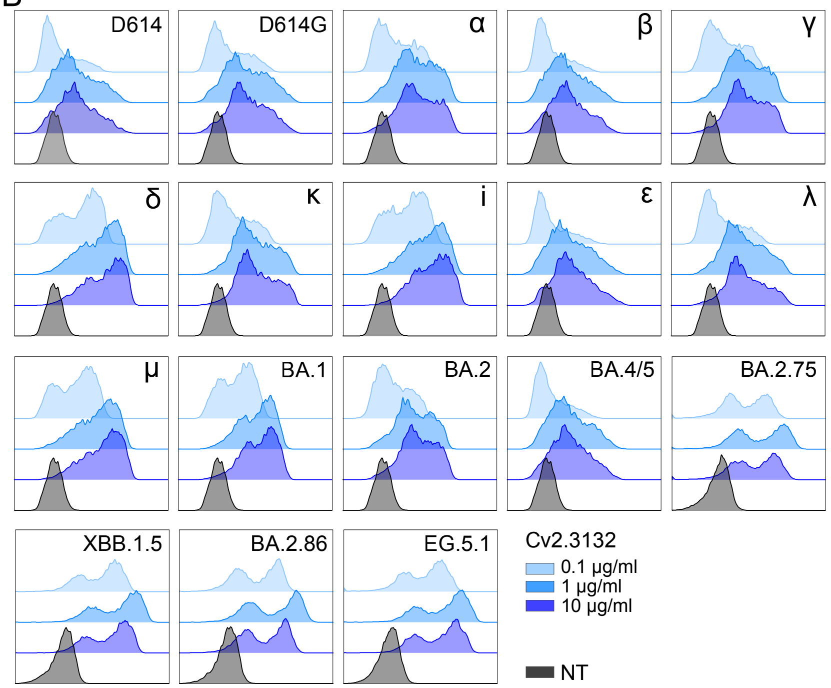

C

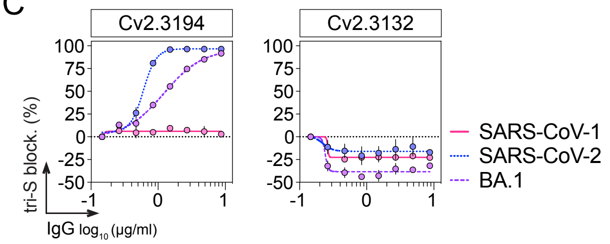

D

Cv2.3194 → ; Cv2.3132 ↓

Cv2.3132 \ Cv2.3194	15	5	1.667	0.556	0.185	0.062	0.021	0.007	0.002	0.001	0.0003	0
150	100	100	100	95	92	77	77	77	76	75	68	70
50	100	100	96	93	89	82	78	71	58	67	67	72
16.667	96	99	100	97	82	82	82	75	69	69	72	71
5.556	100	100	100	96	80	75	72	72	58	61	75	65
1.852	100	100	95	82	71	58	54	60	48	52	46	56
0.617	100	100	94	66	10	48	47	30	28	42	34	41
0.206	100	100	89	34	17	16	1	18	29	42	17	28
0.069	100	99	85	13	22	33	30	27	1	13		15
0.023	97	100	81	32	21	26	32	22	27	7	12	13
0.008	100	99	81	27	23	25	24	15	24	28	25	3
0.003	100	99	77	19		16	10	8	24	5	13	15
0	100	100	77	14	7	10	20	18	16	21	13	22

	150	75	37.5	18.75	9.375	4.6875	2.34375	1.172	0.586	0.293	0.146	0
150	96	96	100	86	59	77	78	56	62	82	70	63
50	91	100	100	63	73	71	61	79	68	77	70	66
16.667	99	95	86	70	76	69	83	79	57	79	71	61
5.556	100	97	91	83	79	75	49	83	60	65	60	59
1.852	95	86	71	77	76	49	72	65	57	55	58	48
0.617	91	95	88	76	66	61	56	59	56	54	31	37
0.206	100	69	77	71	58	37	57	43	22		13	15
0.069	100	81	51	55	55	61	48	33	9	11	0	18
0.023	87	72	58	25	11	16	45	32	17	0	0	16
0.008	73	61	68	42	14	17	25	18	0	39	0	18
0.003	90	73	40	15	0	2	15	0	5	36	5	32
0	95	67	61	38	30	0	2	3	15	0	1	17

Figure S6. Cv2.3132 binding and neutralization properties, related to Figures 5 and 6.
(**A**) Diagram presenting the sequences of the 20-mer overlapping 5-amino acid peptides covering the entire SARS-CoV-2 HR2 region (n=8). The most immunoreactive peptide bound by Cv2.3132 is highlighted in blue.
(**B**) Representative flow cytometric histograms comparing the binding of Cv2.3132 IgG (tested at a concentration 0.1, 1 and 10 µg/ml) to spike-expressing 293-F cells for SARS-CoV-2 variants (SARS-CoV-2, D614G, [VOCs]: α, β, γ, δ, BA.1, BA.2, BA.4/5, BA.2.75, XBB.1.5, BA.2.86, EG.5.1, [VOIs]: ε, ι, κ, λ, µ). NT, non-transfected cell control.
(**C**) Graphs comparing the tri-S-ACE2 blocking capacity of Cv2.3132 IgG with class 1 anti-RBD antibody Cv2.3194 against SARS-CoV-2, BA.1 and SARS-CoV-1 as determined by ELISA. Means ± SD of duplicate values are shown.
(**D**) Dose-response neutralization matrices showing the neutralizing activities of Cv2.3194 and Cv2.3132 alone and combined against BA.4.6 (top) and XBB.1.5 (bottom). IC_{50} values for each combination are indicated in the cells.

Table S1. Data collection and refinement statistics of the RBD-CV2.3194 Fab crystallized complexes, related to Figures 4 and S4.

	RBD^{Wu}+Cv2.3194 (PBD ID: 8QH0)	$RBD^{BA.4/5}$+Cv2.3194 (PBD ID: 8QH1)
**Data collection**		
Space group	$P\ 2_1\ 2_1\ 2_1$	I 1 2 1
Cell dimensions		
a, b, c (Å)	54.4, 89.2, 174.5	57.8, 85.4, 193.99
α, β, γ (°)	90, 90, 90	90, 97.5, 90
Resolution (Å)	39.7-1.9 (1.94-1.87)	39.0-2.65 (2.78-2.65)
Total reflections	394,144 (39,267)	92,060 (12,252)
Unique reflections	70,744 (6,950)	27,213 (3,610)
Completeness (%)	99.5 (98.9)	99.7 (99,9)
Redundancy	13.2 (0.96)	3,4 (3.4)
R_{merge}	0.068 (1.50)	0.094 (1.489)
R_{pim}	0.032 (0.681)	0.060 (0.952)
I/σ (I)	13.2 (0.96)	6.7 (0.6)
$CC_{1/2}$	0.999 (0.613)	0.997 (0.397)
**Refinement**		
Resolution (Å)	39.7-1.9 (1.94-1.87)	39.0-2.65 (2.78-2.65)
No. reflections	70,723 (6,946)	27,187 (2,690)
No. of reflections for Rfree	3,401 (328)	1,402 (139)
R_{work}/R_{free}	0.18 (0.43) / 0.21 (0.47)	0.20 (0.35) / 0.23 (0.37)
No. atoms	5154	4754
Protein and sugar	4730	4732
Ions/Buffer	40	14
Water	384	8
**Mean B value ($Å^2$)**		
Protein and sugar	43.6	79.7
Ligand/Ion	68.2	120.3
Water	47.6	65.0
**R.m.s. deviations**		
Bond lengths (Å)	0.006	0.002
Bond angles (°)	0.82	0.54
Ramachandran favored/outliers (%)	96.9/0.0	96.2/0.2

Statistics for the highest-resolution shell are shown in parentheses.

Table S2. Buried surface area (BSA) at the RBD-Cv2.3194 Fab interface, related to Figures 4 and S4.

	RBD^{Wu} + Cv2.3194 (PDB ID: 8QH0)	RBD$^{BA.4/5}$ + Cv2.3194 (PDB ID: 8QH1)
	BSA PARATOPE	
**Heavy chain**	**740.0 (69.9%)**	**717.6 (69.8%)**
FWR1	20.4 (2.6%)	17.4 (2.4%)
CDRH1	217.4 (29.4%)	250.2 (34.9%)
FWR2		
CDRH2	252.9 (34.2%)	201.1 (28%)
FWR3		37.5 (5.2%)
CDRH3	253.0 (34.2%)	211.3 (29.5%)
FWR4		
**Light chain**	**318.7 (30.1%)**	**310.0 (30.2%)**
FWR1	20.2 (6.3%)	12.5 (4%)
CDRL1	285.8 (89.7%)	277.0 (89.3%)
FWR2		
CDRL2		
FWR3	12.6 (4.0%)	13.1 (4.2%)
CDRL3		6.8 (2.4%)
FWR4		
**TOTAL**	**1058.7**	**1027.6**
	BSA EPITOPE	
**Heavy chain**	**703.5**	**673.4**
**Light chain**	**307.4**	**298.0**
**TOTAL**	**1010.9**	**971.4**

Table S3. Buried amino acid residues at the RBD-Cv2.3194 Fab interface for the different crystallized complexes, related to Figures 4 and S4.

RBD^{Wu} + Cv2.3194 PDB ID: 8QH0		RBD$^{BA.4/5}$ + Cv2.3194 PDB ID: 8QH1	
RBD residue	Fab residue	RBD residue	Fab residue
A: R403	H: V2	E: R403	H: V2
A: T415	H: G26	E: T415	H: G26
A: G416	H: I27	E: G416	H: I27
A: K417	H: T28	E: N417	H: T28
A: D420	H: T30	E: D420	H: T30
A: Y421	H: S31	E: Y421	H: S31
A: G446	H: N32	E: Y453	H: N32
A: G447	H: Y33	E: L455	H: Y33
A: Y449	H: Y52	E: F456	H: Y52
A: Y453	H: P53	E: R457	H: P53
A: L455	H: G54	E: K458	H: G54
A: F456	H: G55	E: S459	H: G55
A: R457	H: S56	E: N460	H: S56
A: K458	H: T57	E: Y473	H: T57
A: S459	H: F58	E: Q474	H: F58
A: N460	H: R97	E: A475	H: R97
A: Y473	H: D98	E: G476	H: L99
A: Q474	H: L99	E: N477	H: V100
A: A475	H: V100	E: V486	H: V101
A: G476	H: V101	E: N487	H: Y102
A: S477	H: Y102	E: Y489	L : I2
A: E484	H: D105	E: F490	L : Q27
A: F486	H: V106	E: Q493	L : S28
A: N487	L : I2	E: S494	L : V29
A: Y489	L : Q27	E: Y495	L: S31
A: F490	L : S28	E: T500	L: S32
A: Q493	L : V29	E: Y501	L: Y33
A: S494	L: S31	E: G502	L: T57
A: Y495	L: S32	E: V503	L: G69
A: G496	L: Y33	E: G504	L: Q91
A: F497	L: S68	E: H505	
A: Q498	L: G69		
A: T500			
A: N501			
A: G502			
A: V503			
A: T505			

Table S4. Polar contacts at the RBD-Cv2.3194 Fab interface for the different crystallized complexes, related to Figures 4 and S4.

RBD^{Wu} + Cv2.3194 PDB ID: 8QH0			RBD$^{BA.4/5}$ + Cv2.3194 PDB ID: 8QH1		
RBD residue	Fab residue	Distance (Å)	RBD residue	Fab residue	Distance (Å)
A: A475 (O)	H: T28 (N)	3.13	E: A475 (O)	H: T28 (N)	3.11
A: A475 (O)	H: N32 (ND2)	2.96	E: A475 (O)	H: S31 (OG)	3.50
A: L455 (O)	H: Y33 (OH)	2.71	E: Q474 (O)	H: S31 (OG)	3.83
A: Y421 (OH)	H: G54 (N)	2.83	E: L455 (O)	H: Y33 (OH)	2.35
A: D420 (OD2)	H: S56 (OG)	2.54	E: Y421 (OH)	H: G54 (N)	2.70
A: N487 (OD1)	H: R97 (NH1)	2.99	E: D420 (OD2)	H: S56 (OG)	2.38
A: Y489 (OH)	H: R97 (NH2)	3.58	E: N487 (OD1)	H: R97 (NH1)	2.25
A: N487 (OD1)	H: R97 (NH2)	3.13	E: N477 (N)	H: G26 (O)	3.48
A: N487 (ND2)	H: G26 (O)	2.72	E: N487 (ND2)	H: G26 (O)	3.47
A: S477 (N)	H: T28 (OG1)	3.68	E: Y473 (OH)	H: S31 (O)	2.68
A: Y473 (OH)	H: S31 (O)	2.60	E: N460 (ND2)	H: G54 (O)	3.40
A: K458 (NZ)	H: S31 (OG)	3.75			
A: K417 (NZ)	H: Y52 (OH)	3.14			
A: Q493 (NE2)	H: Y102 (OH)	3.24			
A: G496 (O)	L: S31 (N)	3.82	E: Y453 (OH)	L: Y33 (OH)	2.77
A: G496 (O)	L: S31 (OG)	3.44	E: R403 (NH2)	L: Y33 (OH)	3.55
A: Y449 (OH)	L: S31 (OG)	3.17			
A: G502 (O)	L: S28 (O)	2.86			
A: N501 (ND2)	L: S28 (O)	3.34			
A: Q498 (NE2)	L: S28 (OG)	3.88			
A: N501 (ND2)	L: S28 (OG)	3.02			
A: Q498 (NE2)	L: S30 (O)	2.76			
A: N501 (ND2)	L: S30 (O)	2.96			
